# First-in-Class Isonipecotamide-Based Thrombin and Cholinesterase Dual Inhibitors with Potential for Alzheimer Disease

**DOI:** 10.3390/molecules26175208

**Published:** 2021-08-27

**Authors:** Rosa Purgatorio, Nicola Gambacorta, Modesto de Candia, Marco Catto, Mariagrazia Rullo, Leonardo Pisani, Orazio Nicolotti, Cosimo D. Altomare

**Affiliations:** Department of Pharmacy—Pharmaceutical Sciences, University of Bari Aldo Moro, Via Orabona 4, 70125 Bari, Italy; rosa.purgatorio@uniba.it (R.P.); nicola.gambacorta1@uniba.it (N.G.); marco.catto@uniba.it (M.C.); mariagrazia.rullo@uniba.it (M.R.); leonardo.pisani@uniba.it (L.P.); orazio.nicolotti@uniba.it (O.N.); cosimodamiano.altomare@uniba.it (C.D.A.)

**Keywords:** acetylcholinesterase, butyrylcholinesterase, Alzheimer’s disease, antithrombotic agents, isonipecotamides

## Abstract

Recently, the direct thrombin (thr) inhibitor dabigatran has proven to be beneficial in animal models of Alzheimer’s disease (AD). Aiming at discovering novel multimodal agents addressing thr and AD-related targets, a selection of previously and newly synthesized potent thr and factor Xa (fXa) inhibitors were virtually screened by the Multi-fingerprint Similarity Searching aLgorithm (MuSSeL) web server. The *N*-phenyl-1-(pyridin-4-yl)piperidine-4-carboxamide derivative **1**, which has already been experimentally shown to inhibit thr with a K_i_ value of 6 nM, has been flagged by a new, upcoming release of MuSSeL as a binder of cholinesterase (ChE) isoforms (acetyl- and butyrylcholinesterase, AChE and BChE), as well as thr, fXa, and other enzymes and receptors. Interestingly, the inhibition potency of **1** was predicted by the MuSSeL platform to fall within the low-to-submicromolar range and this was confirmed by experimental K_i_ values, which were found equal to 0.058 and 6.95 μM for *ee*AChE and *eq*BChE, respectively. Thirty analogs of **1** were then assayed as inhibitors of thr, fXa, AChE, and BChE to increase our knowledge of their structure-activity relationships, while the molecular determinants responsible for the multiple activities towards the target enzymes were rationally investigated by molecular cross-docking screening.

## 1. Introduction

Alzheimer’s disease (AD) is a devastating neurodegenerative disorder, which accounts for the most elderly-related dementias. It is estimated that more than ten million people in Europe are currently affected by AD and other dementias, and this number is expected to increase to over thirteen million in 2030 [1,2].

The main histopathological hallmarks of AD are deposition of extracellular neurotoxic amyloid-β (Aβ) peptide aggregates, along with intracellular neurofibrillary tangles of hyperphosphorylated τ-protein, that generates senile plaques and trigger oxidative stress along with perturbation of cellular metabolism, finally leading to synaptic and neuronal loss. In addition, the lowering of the level of cholinergic neurotransmitter acetylcholine (ACh) into the hippocampus, and progressively into the whole brain cortex, contributes to the typical AD-related cognitive and memory impairment and decline [2,3,4].

Despite the efforts devoted to discovering novel disease-modifying pharmacological treatments, only few drugs are available for symptomatic treatment of mild-to-moderate AD, which are the acetylcholinesterase (AChE) reversible (galantamine and donepezil) or pseudo-irreversible (rivastigmine) inhibitors, and the *N*-methyl-d-aspartate receptor (NMDAR) antagonist memantine.

A relevant number of elderly persons affected by AD report comorbidities, mostly cardiovascular conditions and/or diabetes. Vascular and thrombotic risk factors (i.e., hypertension, diabetes, and hyperlipidemia) have also been described as potentially associated with an increased risk and progression of cognitive decline in AD and vascular and mixed dementia [5]. Depending upon the disease severity, stroke is three-to-seven times more common in these patients, and the cerebrovascular lesions may lower the threshold for clinical manifestation of AD. As recommended by recent medical guidelines, the systematic prevention of vascular risk factors in AD patients may positively impact the disease progression [6]. The cerebrovascular abnormalities are followed by formation of Aβ protein plaques co-aggregating with some coagulation factors; these deposits may induce leaking of the brain-blood-barrier (BBB), promoting a pro-thrombotic state, as well as enhancing release of pro-inflammatory mediators in the brain areas [7].

**Figure 1:**
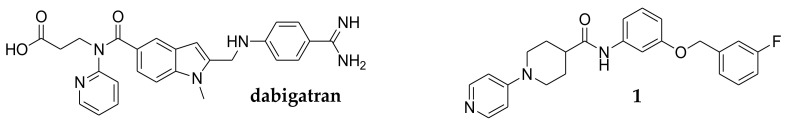
Dabigatran and the in vitro equipotent isonipecotamide-based thrombin inhibitor **1**.

Early clinical trials with small groups of patients showed positive effects of treatments with anticoagulant drugs in dementia-related disorders. Beneficial effects in AD mouse models have been observed after long- and short-term administration of the clinically available thrombin (thr) inhibitor dabigatran (Figure 1), which belongs to the family of direct oral anticoagulants (DOACs).

Thrombin is a pleiotropic protease that, as well as its well-known functions in coagulation cascade, has been recognized to play a role in AD as a key inflammatory mediator in the brain. It has also been suggested that vascular injuries associated with neurodegenerative diseases could increase the permeability of BBB to thrombin, which can finally activate protease activated receptor (PAR) expressed on microglia and astrocytes, thus enhancing the AD-associated neurotoxicity and inflammation [8,9].

Treatment with dabigatran resulted in mitigation of neuroinflammation, reduction of the histopathological AD hallmarks, and ultimately in the recovery of cognitive decline [10]. To date, the patients’ recruiting step has started to test efficacy of dabigatran in ameliorating physiopathology and cognitive decline in AD in a repositioning phase I clinical trial [11].

In the last decade we reported the rational design of several classes of (iso)nipecotamide derivatives, acting as selective thrombin (thr) or factor Xa (fXa) inhibitors [12,13,14,15,16]. A pool of these compounds was screened by employing a new upcoming release of the Multi-fingerprint Similarity Search aLgorithm (MuSSeL), an in-house web server recently developed by some of us [17,18] in an attempt to find other putative and clinically relevant targets. Interestingly, among the screened compounds, the isonipecotamide derivative **1** (Figure 1) [15] was flagged by MuSSeL as a putative binder of several AD-related protein targets, which include a number of heterologous cholinesterases (e.g., electric eel AChE and equine butyrylcholinesterase BChE).

ChE inhibitors, arising from the “old” cholinergic hypothesis, currently remain the few treatments available to AD patients. Starting from seminal papers on the therapeutic potential of multi-target directed ligands (MTDLs) as disease-modifying agents [19,20], many efforts have been reported to identify new MTDLs, mostly by conjugating AChE inhibitors (donepezil and/or the withdrawn drug tacrine) with a molecular moiety able to interact with additional targets such as Aβ and τ protein aggregation or involved in balancing oxidative stress [21,22].

Inspired by the MTDL paradigm, herein we aimed at integrating in one molecule the potential to bias cholinesterases (ChEs), on one side, and key coagulation factors, thr and/or fXa, on the other. The predicted anticholinesterase activity of compound **1** was experimentally investigated. Furthermore, by selecting a set of structurally related compounds, the molecular determinants responsible of the multitarget activity were rationally investigated by molecular cross-docking screens.

## 2. Results

### 2.1. Target Protein Prediction by Similarity Search

**Figure 2:**
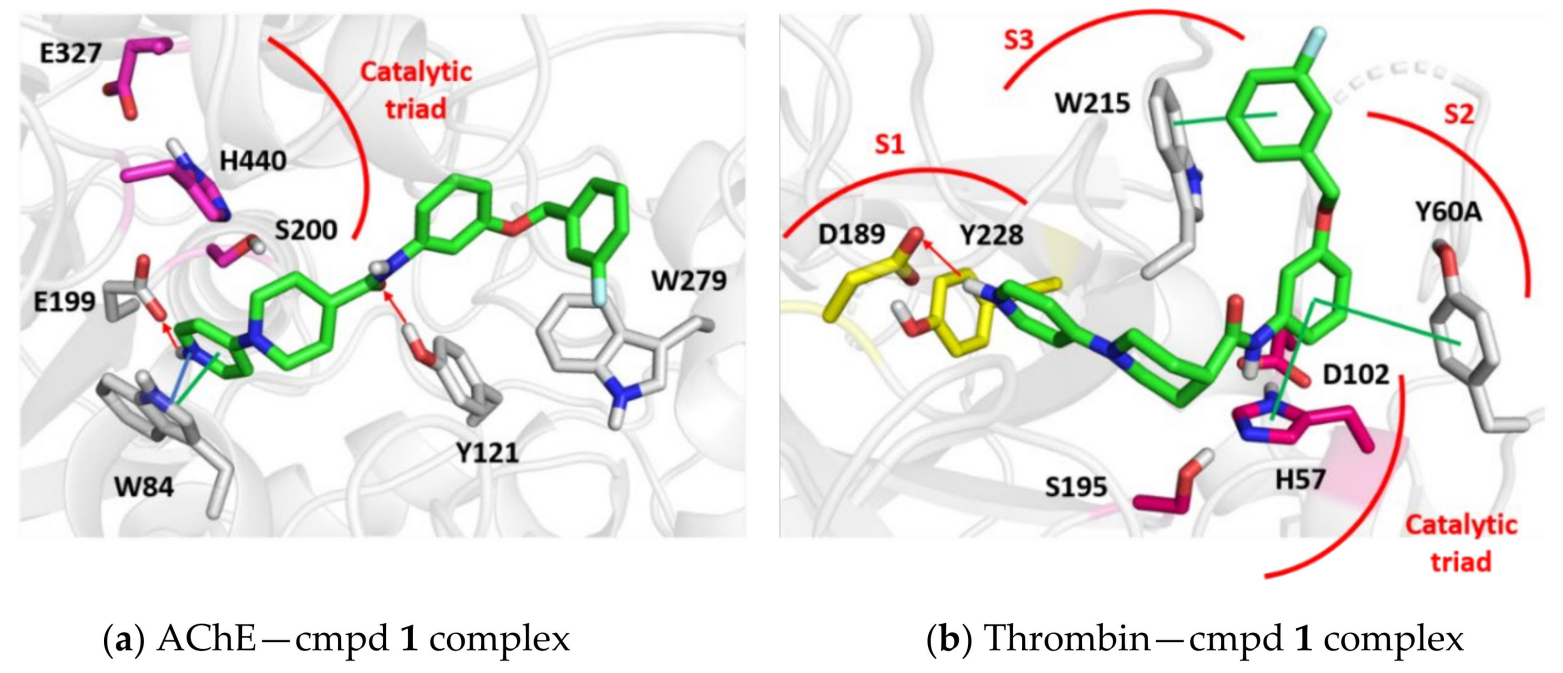
Top-scored docking poses of cmpd **1** vs. AChE (*T. Californica*, PDB_ID: 1EVE) and vs. thr (*H. Sapiens*, PDB_ID: 1UVS) are shown on the left and right hand-side, respectively. The protein is shown in ribbon, and the ligands and potentially interacting residues are shown in stick format. Catalytic triads are colored in magenta. Green and blue lines report π–π and cation–π interactions. Red arrows indicate hydrogen bonds. Pockets S1, S2 and S3 in the thr binding site are flagged in red. Docking score values are equal to −8.613 and −8.718 kcal/mol for **1** vs. AChE and thr, respectively.

Based on multi-fingerprint similarity analyses, MuSSeL characterized compound **1** as a privileged structure sharing both evident and latent molecular frameworks with many known bioactive compounds, thus returning **1** as a potential hit biasing a number of relevant protein drug targets (see Supporting Information), including ChEs (both AChE and BChE), without significative differences between human and heterologous ChE isoforms. A computational study was carried out to investigate at a molecular level the putative interactions established by **1** towards active sites of AChE and thr (Figure 2).

Thr and fXa are trypsin-like serine proteases that in sequence catalyze the conversion of soluble fibrinogen into insoluble fibrin in the final step of clot formation. The thrombin active site shows the serine proteases’ catalytic triad (residues Asp102, His57, and Ser195), and four binding subsites (S1−S4) recognizing substrate accommodation [15]. The S1 subsite is a deep hydrophobic pocket, that can be considered the major determinant of substrate specificity; the Asp189 residue at the bottom of S1 shows a preference for the Arg residue of protein substrates. A proximal S2 pocket (the so-called loop 60) accommodates small-size hydrophobic residues. A flat S3 site may bind substrates in an antiparallel hydrogen bonding pattern usually involving Gly216. Finally, a large hydrophobic S4 site, also called aryl binding site, and lined by hydrophobic residues (Leu99, Ile174, and Trp215) interacts with aromatic residues or large aliphatic moieties. FXa shares high homology with thrombin, mostly in the S1 subsite, thus laying the main differences in the absence of S2 subsite and in the different residues lining the S4 aromatic subsite, where Tyr99 and Phe174 replace the corresponding Leu and Ile residues of thrombin.

ChEs are serine proteases that hydrolyze the neurotransmitter acetylcholine (ACh). AChE is the main enzyme responsible for ACh regulation in the healthy brain. In the AD patient brain, a decline of AChE level and a significant increase (from 30 to 60%) of BChE expression and activity have been reported [23,24], suggesting a role of BChE in AD progression. AChE and BChE share about 70% of structural homology. The AChE active site is a 20 Å deep and narrow gorge, containing five regions which recognize the binding of ligands, namely: the catalytic active site (Ser203, His447, Glu334, human species numbering); the ‘oxyanion hole’, that stabilizes the transient tetrahedral enzyme-substrate complex; the ‘anionic site’ where the residue Trp86 (conserved in both ChEs) recognize, through cation–π interactions, orientation and stabilization of the trimethylammonium head of ACh; the ‘acyl pocket’ interacting with the substrate acyl group; and the so-called ‘peripheral anionic site’ (PAS), located on the rim of the active site gorge [23,24]. BChE differs from AChE in the ‘acyl pocket’ and PAS. Two Phe residues (Phe295, Phe297) in the AChE ‘acyl pocket’, limiting the access of bulkier molecules to the catalytic site, are replaced by two aliphatic residues (Leu286, Val288) in BChE. Furthermore, six out of the fourteen aromatic residues lining the AChE gorge rim and PAS are replaced by aliphatic residues in BChE. Therefore, the volume of the BChE cavity (about 200 Å^3^) is significantly larger than that of AChE.

As shown in Figure 2, the isonipecotamide derivative **1** can establish both π–π and cation–π interactions with the AChE residue Trp84 at the catalytic anion site (CAS), through its pyridyl ring, which is also involved in a hydrogen bond with the side chain of Glu199. The carbonyl group of the isonipecotanilide moiety could make a hydrogen bond with the side chain of Tyr121, while the meta-fluorine substituted benzyloxy group faced to the residue Trp279 at the peripheral anionic site (PAS). As far as the interactions with thrombin are concerned, the pyridine ring can make a hydrogen bond (HB) with residue Asp189 at the S1 pocket. In addition, the aromatic ring of the isonipecotanilide moiety can make π–π contacts with Tyr60A at S2 pocket [25], and with His57 of the catalytic triad [26], while the meta-fluorine benzyloxy group established π–π contacts with Trp215 at the S3 pocket.

Taking into account the predictions, the inhibitory activity of cmpd **1** toward electric eel (ee) AChE and horse serum (hs) BChE was investigated by using the in vitro Ellman colorimetric assay, as modified [27]. The inhibition constant values were derived from IC_50_ by applying the Cheng–Prousoff equation [28].

Compound **1** showed good and selective inhibition of eeAChE (Table 1, K_i_ = 58.0 ± 1.4 nM), affording identification of a new potential MTDL against neurodegeneration, combining in vitro a thr inhibition potency equal to that of dabigatran, and anti-AChE activity quite similar to that of donepezil. The experimental ΔΔG score was about 1.3 kcal/mol, one unit higher than that predicted by the docking study.

**Figure 3:**
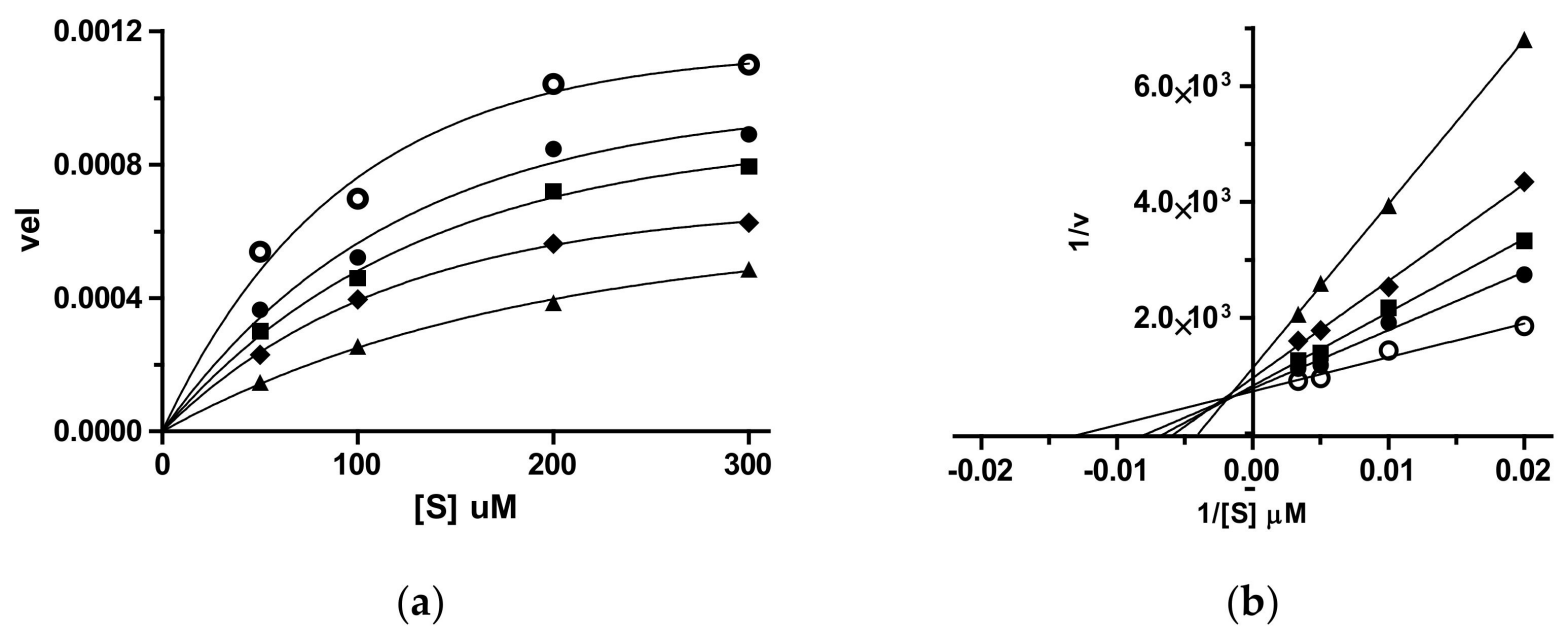
Inhibition kinetics (**a**) and Lineweaver–Burk plot (**b**), r^2^ = 0.966–0.996 for *ee*AChE (0.09 U/mL) and **1** (0–500 nM) by using different substrate (acetylthiocholine iodide) concentrations (50–300 μM). The replot (r^2^ = 0.990) of the slopes versus [I] determined the K_i_ (58 nM) as the *x*-axis intercept (see Supplementary Information). (○) no inhibitor, (●) 50 nM, (■) 100 nM, (◆) 200 nM, (π) 500 nM.

The mechanism of eeAChE inhibition by cmpd **1** was studied (Figure 3). The Lineweaver–Burk curves were outlined using a fixed amount of eeAChE and varying substrate concentrations (50–300 μM), in the absence or presence of different inhibitor concentrations (0–500 nM). Binding of **1** to AChE changed both V_max_ and K_M_ values, by following a trend that can be ascribed to a noncompetitive/mixed-type inhibition. A replot of the slopes versus the corresponding inhibitor concentrations (see Figure S1 in Supplementary Information) provided a K_i_ value of 0.056 μM, superimposable to that obtained by applying the Cheng–Prousoff equation (0.058 μM). The noncompetitive/mixed inhibition mechanism on AChE suggests that the inhibitor may interact with binding sites other than the active site. In our case, the highly scored docking poses suggested that cmpd **1**, with its meta-F-benzyloxy moiety, may bind into the PAS (Trp279) of AChE (Figure 2a).

Based on the dual activity achieved by the isonipecotamide derivative **1,** a wider set of structurally related compounds, previously reported and newly synthesized, showing moderate-to-potent inhibition of thr, were evaluated as ChEs’ inhibitors.

### 2.2. Chemistry

**Scheme 1:**
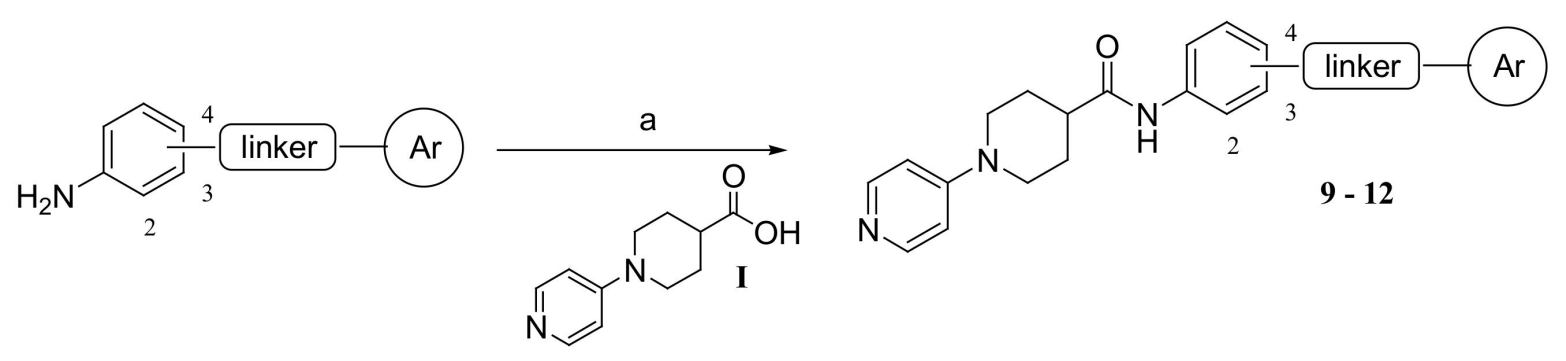
(a) 1-(pyridin-4-yl)-piperidine-4-carboxylic acid (**I**), TBTU, DIPEA, dry DMF, r.t., 48–72 h.

**Scheme 2:**
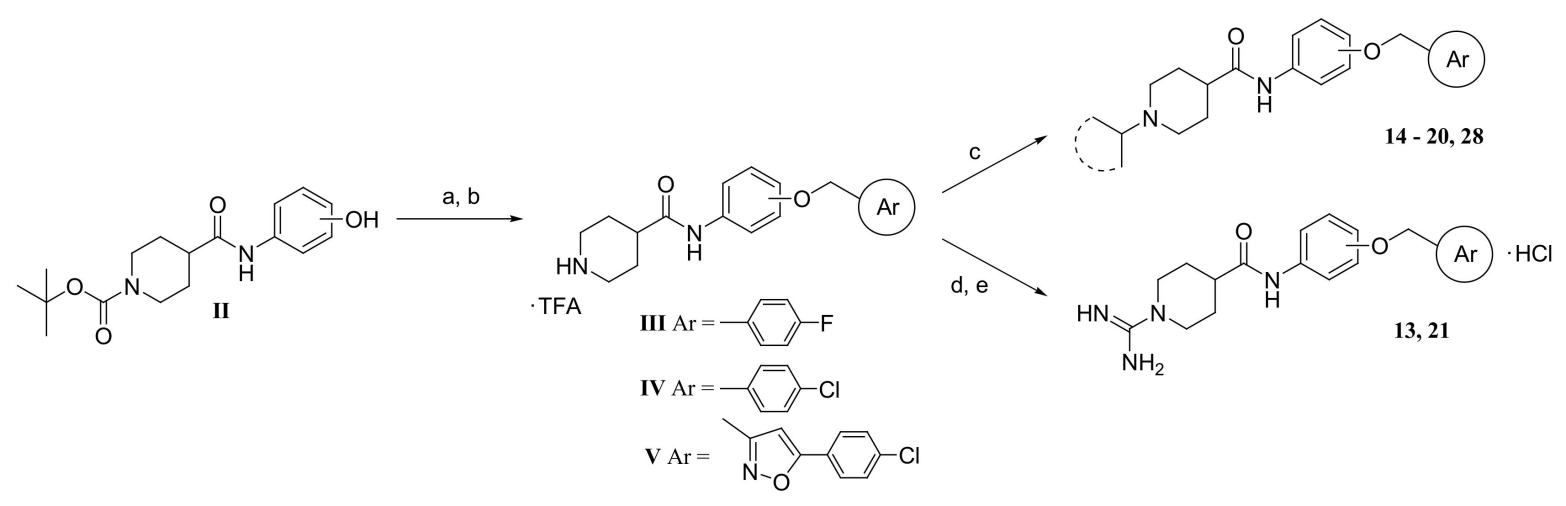
(a) NaH, Ar-CH_2_-X, dry DMF, r.t., 4 h; (b) CH_2_Cl_2_, CF_3_COOH, r.t., 2 h; (c) acetone or cyclopentanone, Na(CN)BH_3_, MeOH, overnight; (d) 1,3-bis(tertbutoxycarbonyl)-2-methyl-2-thiopseudourea, HgCl_2_, dry DMF, 0 °C to r.t., 24 h; (e) 4M HCl in dioxane, CHCl_3_, 0 °C, 2 h.

The newly synthesized compounds **9**–**12** were prepared following methods previously reported for the synthesis of compounds **1**–**8**, as shown in Scheme 1 and Scheme 2 [15]. Using TBTU/DIPEA as coupling reagents in dry DMF, the isonipecotamide derivatives **1**–**12** were prepared in satisfactory yields by reaction with 1-(pyridin-4-yl)piperidine-4-carboxylic acid (**I**), which in turn was obtained in a one-pot reaction between ethyl isonipecotate and 4-chloropyridine hydrochloride, and suitable aniline intermediates [15].

Compounds **13**–**21** were synthesized as shown in Scheme 2 [13]. The N-BOC protected (ortho-, meta-, para-substituted) phenol compounds (**II**) were alkylated with fluoro- and chlorobenzyl, or suitable bromomethyl-biaryl intermediates. After removal of the BOC-protecting group with TFA, the salts **III**–**V** underwent reductive amination with acetone or cyclopentanone, to afford, after chromatographic purification on silica gel, the N-alkyl isonipecotanilides **14**–**20** and **28** [13].

Alternatively, reaction with 1,3-bis(tert-butoxycarbonyl)-2-methyl-2-thiopseudourea and HgCl_2_ in dry DMF, followed by BOC deprotection, afforded HCl salts of the guanidine derivatives **13** and **21** [12,13].

### 2.3. Dual Cholinesterase/Coagulation Factor Inhibition by Isonipecotamide Derivatives

Each compound was first tested at 10^−5^ M concentration, and the compounds achieving at least 60% inhibition were then tested at lower scalar concentrations to determine their half-maximal inhibitory concentrations (IC_50_), and their inhibition constant values (Table 1).

The effects determined by the shift of the F atom, and definition of the better positional isomerism of the OCH_2_ linker between the isonipecotamide scaffold and distal benzyl fragment were investigated. Both inhibitory activities were significantly affected by the position of F. The shift of the F atom from meta to para position retained the dual activity profile in **2** but showed significantly weaker potencies. As previously reported, the meta-F improved selectivity toward thr over fXa, and increased nearly 35-fold the potency against thr over the 4′-F isomer. The loss in activity due to the F-shift was higher against thr than against AChE (cmpd. **2** was four times less active than **1** as AChE inhibitor).

The positional ortho (**4**) and para (**5**) congeners of **1** clearly showed that the meta isomer does achieve higher affinity in both thr and AChE binding sites. The para isomer **4** appeared 2–3 orders of magnitude less potent than **1** against both thr and AChE. The ortho isomer **5** did not show any significant inhibition at the highest tested concentration. Noteworthily, both para and ortho positional isomers appeared as moderate BChE inhibitors, more active than **1**, similarly to some picolinamide derivatives reported by others [29].

The chemistry of the linker connecting distal 4-F-phenyl with nipecotanilide scaffold, previously reported as a key factor to achieve a good thrombin inhibition, was further investigated. Despite the OCH_2_ spacer proving to be the only fragment affording potent thr inhibition, the anti-AChE activity was influenced to a lesser extent by the changing chemistry and physicochemical properties (lipophilicity, polar surface area, H-donor/acceptor properties) of the linker. Derivatives **3** and **7**–**12** showed a quite similar and selective AChE inhibition.

The AChE inhibition potency decreased three times due to a reversal of the oxymethylene OCH_2_ to CH_2_O bridge (**3** vs. **2**); its elongation to OCH_2_CH_2_ (**6** and **7**), or its replacement with CH_2_CH_2_ (**8**) resulted in less effective inhibitors. However, the OCH_2_CH_2_ linker led us to identify an alternative route to integrate in one molecule the potential of interfering with both blood coagulation factors and cholinesterases, thus providing cmpd. **7** with selectivity against fXa and moderate AChE inhibition potency.

The carboxamide (**9**–**10**) and sulphonamide (**11**) linkers resulted in promising AChE inhibitors, which showed a potency quite similar to that of **2**, and a limited loss in potency with respect to **1**. In contrast, the urea linker (**12**) turned out to be unfavorable in all the tested enzymes.

An attempt at correlating the anti-AChE potency with physicochemical descriptors of lipophilicity, molecular volume, molar refractivity, and topological surface area (tPSA), suggested a trend of nonlinear relationships with tPSA, with the property value of cmpd. **1** (54.46 Å^2^) near the optimum. A tPSA value less than 90 Å^2^, according to Clark’s equation, is accepted as a molecular determinant allowing the crossing of the brain-blood-barrier (BBB) and delivering drugs into CNS [30,31]. As simulated in silico admetSAR software, cmpd. **1** was estimated to be able to cross the BBB, be non-hepatotoxic, and be well absorbed through human intestine [32].

The enzymes’ inhibition profile of some 4′-F and 4′-Cl-benzyloxy derivatives, obtained by replacing the pyrid-4-yl moiety on piperidine fragment was further investigated.

The compounds in Table 2 showed only a promising inhibition of ChEs, whilst lacking activity against thr and/or fXa, with the exception of **20**. Substituents on N1 deeply affected anti-ChE activities. While pyridyl moiety on the piperidine N1 identified selective AChE inhibitors, derivatives **13**–**15**, bearing guanidine, alkyl or cycloalkyl groups proved to be selective inhibitors of BChE, with moderate to good potency. The guanidino derivative **13** inhibited BChE about five times more than cmpd **2**; its replacement with isopropyl (**14**, K_i_ = 130 ± 4 nM) or cyclopentyl (**15**, K_i_ = 115 ± 2 nM) further improved potency and selectivity toward BChE. The inhibition mechanism towards BChE was found to be noncompetitive/mixed (Figure S2 in S.I.).

Regarding the positional isomers on the carboxamide scaffold in this set of 4′-F-benzyl derivatives, meta-substituted analogs were more potent than the ortho- and para-substituted isomers. Replacement of F with Cl (compare the *N*-isopropyl derivatives **18** and **20**) enhanced the potency against both BChE and fXa. Compound **20** was flagged as a potential BChE/fXa dual inhibitor, showing K_i_ values toward both enzymes in the same concentration range.

Replacing the *N*-isopropyl group with a guanidine one resulted in a dramatic loss of both BChE and fXa inhibitory activities. The behavior of cmpd. **20**, which showed the highest dual fXa/BChE inhibition potency, was not totally unexpected, as it could adopt the so called L-shape conformation, typical of the most potent fXa inhibitors, thus allowing the halogen-binding mode [33] between the chlorophenyl fragment and S1 subsite [13,14]. Furthermore, **20** could well access the BChE binding site, due to the gorge larger than that of AChE.

The achievement in the same molecule of a good fXa and BChE inhibition must not be underestimated. A putative role of BChE in the AD progression has been suggested [23,24], whereas, although a direct involvement of fXa in abnormal neurodegenerative pathways has not been shown, fXa inhibition can prevent the generation of thr and possibly its AD-related neurotoxicity and inflammation [6,7].

An in-house library of isopropyl- and guanidino isonipecotamide fXa inhibitors, with nanomolar potency, bearing a biaryl moiety, was further evaluated for the inhibitors additional abilities as ChE inhibitors. The derivatives in Table 3 showed weak potency as ChEs’ inhibitors, with the exception of **23**, bearing a guanidino group at piperidine nitrogen and 4-Cl-biphenyl moiety, which showed a good dual fXa/BChE inhibition potency in the nanomolar range (fXa K_i_ = 31 ± 1 nM; BChE K_i_ = 90 ± 4 nM). The corresponding *N*-isopropyl analogue **22** did not show better fXa and BChE inhibition. Compounds obtained by replacing 4-Cl-biphenyl with bioisosteric phenylisoxazoles, previously reported as enhancers of fXa inhibition potency, were two orders of magnitude less active than **22** and **23** towards BChE.

**Figure 4:**
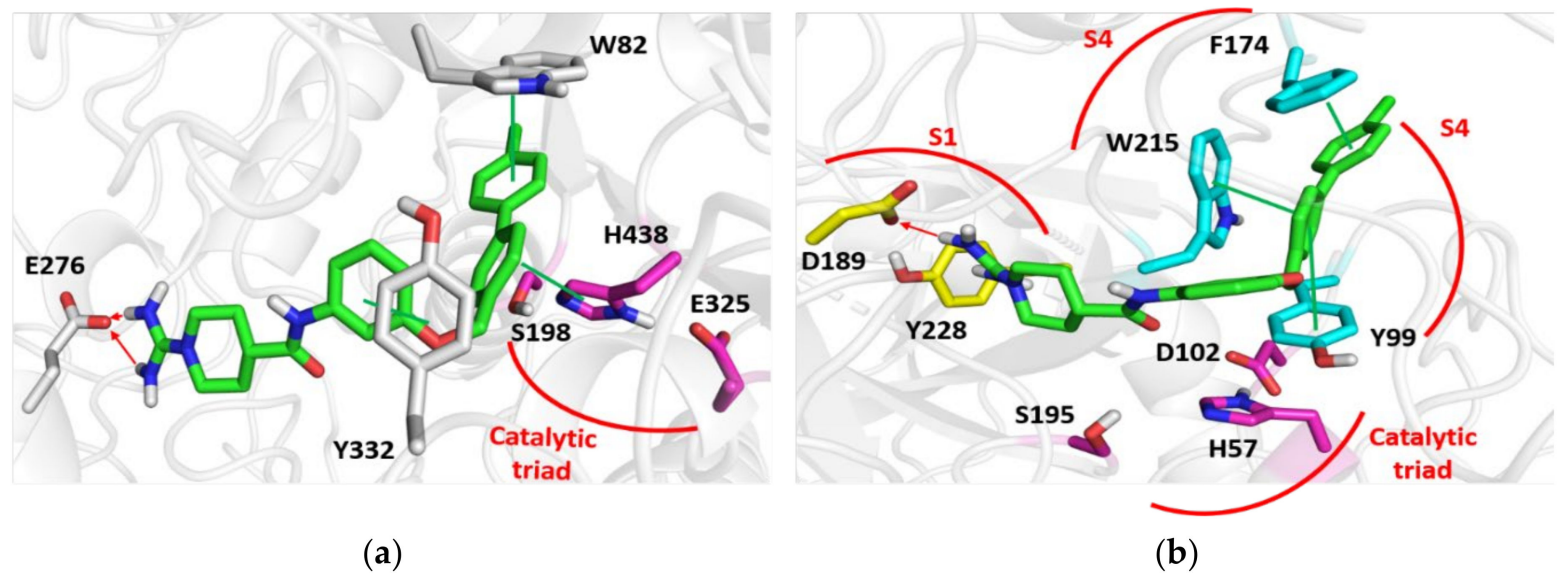
Top-scored docking poses of compound **23** vs. BChE (*H. Sapiens,* PDB_ID: 6SAM) and vs. fXa (*H. Sapiens*, PDB_ID: 3K9X) are shown on the left (**a**) and right (**b**) hand-side, respectively. Catalytic triads are colored in magenta. Green lines and red arrows indicate π–π interactions and hydrogen bonds, respectively. Pockets S1 (yellow sticks) and S4 (cyan sticks) in the fXa binding site are flagged in red. Docking score values were equal to −9.861 and −10.663 kcal/mol for **23** vs. BChE and vs. fXa, respectively.

A molecular docking study on human BChE, which shares high structural homology with horse serum isoform, was carried out to investigate molecular interactions of cmpd. **23** and BChE (Figure 4). As shown on the left-hand side of Figure 4, **23** can make a hydrogen bond with the side chain of Glu276 residue, through its guanidine group. Interestingly, a π–π interaction was detected between the aromatic ring of isonipecotanilide and Tyr332, and between the para-Cl biphenyl group with Trp82 and His438, the latter being part of the catalytic triad. As far as fXa interactions were concerned, **23** established a hydrogen bond with Asp189 at the S1 pocket through its guanidine group. Interestingly, the para-Cl biphenyl arm can strongly interact through π–π contact with residues Trp215, Phe174, and Tyr99 at the S4 fXa selective pocket.

**Figure 5:**
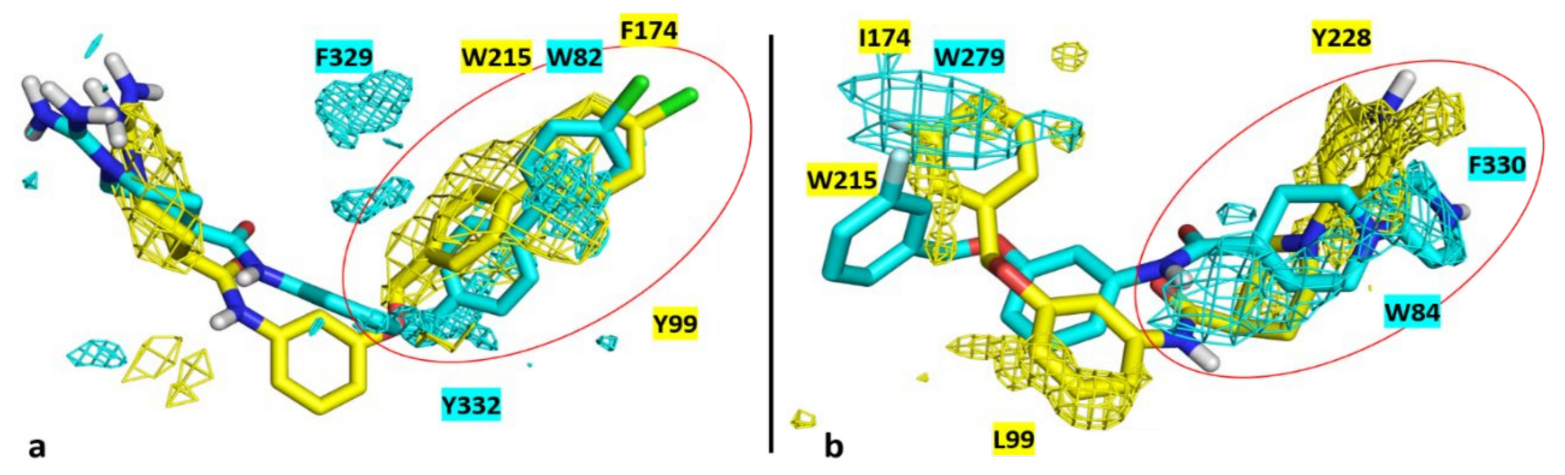
Panel (**a**) shows the structural overlays of **23** within BChE and fXa binding pockets; panel (**b**) shows the structural overlays of **1** within AChE and thrombin binding pockets. Cyan/yellow mesh indicates AChE/BChE and fXa/thr lipophilic MIFs detected by CRY probe. The most relevant residues are highlighted in cyan for AChE and BChE and in yellow for fXa and thr.

To investigate the molecular determinants responsible for the observed multitarget activities, the rational analysis of cavities was performed, by focusing on compounds **1** and **23**, which were proved to target with the highest (nanomolar) binding affinity thr/AChE and fXa/BChE, respectively (Figure 5). The structural alignment of top-scored docking poses of **1** towards AChE and thr, and of **23** towards BChE and fXa was carried out to make results easily comparable. Interestingly, cmpd. **1** adopted a more bent conformation within the thr binding site and a more relaxed arrangement within the AChE binding pocket. On the other hand, compound **23** assumed very similar L-shaped (or V-shaped) conformations within the two binding pockets. 

The BioGPS software was employed to compute the molecular interaction fields (MIFs) in an attempt at identifying common features between the binding pockets. In this respect, the MIFs similarity between BChE and fXa, and AChE and thr are reported in Supplementary Information (Tables S1 and S2).

Looking at Figure 5, the *para*-Cl biphenyl ring of **23** impacted overlapping regions of minimum CRY probe formed by Trp82 and Tyr332 residues of BChE, and by Phe174, Trp215, and Tyr99 residues of fXa. As for AChE and thr cavities, the 4-(piperidin-1-yl)pyridine moiety of **1** lay in a CRY minimum region formed by Trp84 and Phe330 residues of AChE and Tyr228 residue of thrombin. Furthermore, the *meta*-fluorine benzyloxy group impacted another CRY minimum made up of Trp279 residue of AChE and Ile174 and Trp215 residues of thr.

## 3. Materials and Methods

### 3.1. Chemistry

For the starting materials, all chemicals and solvents were purchased from Sigma-Aldrich and Alfa Aesar. Melting points were determined by using the capillary method on a Stuart Scientific SMP3 electrothermal apparatus and are uncorrected. Purity of the final compounds was assessed by elemental analyses (C, H, N), performed on a Euro EA3000 analyzer (Eurovector, Milan, Italy), by the Analytical Laboratory Service of the Department of Pharmacy-Drug Sciences of the University of Bari Aldo Moro (Italy). The results agreed to within 0.4% of theoretical values. All the tested compounds showed a higher than 95% purity. Mass spectra were obtained by Agilent 1100 Series LC–MSD Trap System VL, equipped with ESI (electrospray ionization) source. The high-resolution molecular masses of test compounds were assessed by Agilent 6530 Accurate Mass Q-TOF spectrometer (Agilent Technologies, Santa Clara, CA, USA). IR spectra (KBr disks) were recorded on a Spectrum One FT infrared spectrophotometer (PerkinElmer, Beaconsfield, UK), and the most significant absorption bands are listed. ^1^H NMR spectra were recorded at 300 MHz on a Varian Mercury 300 instrument. Chemical shifts data are expressed in δ and the coupling constants J are in hertz (Hz); the following abbreviations are used for multiplicity: s, singlet; d, doublet; dd, doublet-doublet; td, triplet of doublets; t, triplet; q, quartet; m, multiplet. Signals due to NH and OH protons were located by deuterium exchange with D_2_O. Chromatographic separations were performed on silica gel 60 for column chromatography (Merck 70–230 mesh).

#### 3.1.1. Synthesis of Compounds **9–12**

Compounds **9**–**12** were synthesized by following the procedure previously reported [15]. To a solution of 205 mg (1.00 mmol) of 1-(pyridin-2-yl)piperidine-4-carboxylic acid (**I**) in 10 mL of dry DMF were added 320 mg (1.00 mmol) of TBTU, 0.68 mL (4.00 mmol) of DIPEA, and, after 30 min, the suitable aniline intermediate (1.2 mmol) was added. The reaction mixture was stirred at room temperature for 72 h and then poured on ice. The resulting precipitate was filtered, collected, and washed with diethyl ether (Et_2_O) to yield the desired compounds. When necessary, the crude products were recrystallized from acetone and MeOH.

*N-(3-(4-fluorophenylcarbamoyl)phenyl)-1-(pyridin-4-yl)piperidine-4-carboxamide* (**9**). Brown solid, yield 55% (230 mg), M.p. 130–132 °C. IR cm^−1^: 3449, 1669, 1604, 1509, 1484, 1434, 1232, 1188, 998, 806, 751. ^1^H NMR (300 MHz, acetone-d_6_), *δ*_H_: 9.65 (s, 1H), 9.40 (s, 1H), 8.28–8.13 (m, 2H), 7.95–7.81 (m, 2H), 7.65 (d, 1H, *J* = 8 Hz), 7.13 (t, 2H, *J* = 8 Hz), 6.82 (d, 2H, *J* = 6.5 Hz), 2.98 (dt, 2H, J1 = 12.5 Hz, J2 = 2.5 Hz), 2.85–2-70 (m, 1H), 2.05–1.99 (m, 2H), 1.88–1.78 (m, 2H). HRMS [ESI], *m*/*z*: 419.1868 [M + H]^+^ for C_24_H_24_N_4_O_2_F. Anal. Calc. for C_24_H_23_N_4_O_2_F, %: C, 68.88; H, 5.54; N, 13.39; found, %: C, 68.98; H, 5.75; N, 13.42.

*N-(3-(4-fluorobenzylcarbamoyl)phenyl)-1-(pyridin-4-yl)piperidine-4-carboxamide* (**10**). Pale brown solid, yield 56% (240 mg), M.p. 148–150 °C. IR cm^−1^: 1669, 1604, 1509, 1484, 1434, 1232, 1188, 998, 806, 751. ^1^H NMR (300 MHz, acetone-d_6_), *δ*_H_: 9.38 (br s, 1H), 8.29 (br s, 1H), 8.22–8.12 (m, 2H), 7.84 (dd, 2H, *J* = 8.3, 1.1 Hz), 7.59 (d, 1H, *J* = 8.0 Hz), 7.44–7.34 (m, 4H), 7.07 (t, 1H, *J* = 8.8 Hz), 6.80 (d, 2H, *J* = 6.5 Hz), 4.58 (d, 2H, *J* = 5.8 Hz), 4.03 (d, 2H, *J* = 13 Hz), 2.99–2.76 (m, 2H)2.74–2.66 (m, 1H), 2.06–1.90 (m, 2H), 1.86–1.75 (m, 2H). HRMS [ESI], *m*/*z*: 433.2029 [M + H]^+^ for C_25_H_26_N_4_O_2_F. Anal. Calc. for C_25_H_25_N_4_O_2_F, %: C, 69.43; H, 5.83; N, 12.95; found, %: C, 69.45; H, 5.87; N, 13.00.

*N-(3-(4-fluorobenzylsulphonyl)phenyl)-1-(pyridin-4-yl)piperidine-4-carboxamide* (**11**). Brown solid, yield 50% (235 mg), M.p. 185–188 °C. IR cm^−1^: 3341, 2926, 2856, 1630, 1600, 1540, 1510, 1324, 1310, 1221, 1151, 993, 788. ^1^H NMR (300 MHz, DMSO-d_6_), *δ*_H_: 10.24 (s, 1H), 8.21–8.08 (m, 2H), 7.93 (t, 1H, *J* = 6.3 Hz), 7.75 (d, 1H, *J* = 8.0 Hz), 7.40 (dt, 1H, *J* = 6.3, 4.4 Hz), 7.31–7.23 (m, 2H), 7.16 (t, 1H, *J* = 7.3 Hz), 7.13–7.02 (m, 2H), 6.91–6.69 (m, 2H), 5.54 (s, 1H), 4.05–3.85 (m, 4H), 2.94–2.87 (m, 2H), 2.69–2.54 (m, 1H), 1.87 (d, 2H, *J* = 10.6 Hz), 1.65 (t, 2H, *J* = 13 Hz). HRMS [ESI], *m*/*z*: 469.1699 [M + H]^+^ for C_24_H_26_N_4_O_3_SF. Anal. Calc. for C_24_H_25_N_4_O_3_SF, %: C, 61.52; H, 5.38; N, 11.96; found, %: C, 61.77; H, 5.43; N, 12.01.

*1-(3-(1-(pyridin-4-yl)piperidine-4-carboxamido)phenyl)-3-(4-fluorophenyl)urea* (**12**). Pale brown solid, yield 75% (325 mg), M.p. 229–231 °C. IR cm^−1^: 3288, 1638, 1604, 1510, 1491, 1446, 1296, 1202, 998, 838, 809, 688.^1^H NMR (300 MHz, DMSO-d_6_), *δ*_H_: 9.92 (s, 1H), 8.68 (s, 1H), 7.48 (s, 1H), 8.33–8.10 (m, 2H), 8.10 (d, 2H, *J* = 2.0), 7.78 (s, 1H), 7.45–7.38 (m, 2H), 7.22–7.06 (m, 3H), 6.85–6.72 (m, 2H), 4.00–3.98 (m, 2H), 2.94–2.82 (d, 2H, *J* = 12.4 Hz), 2.49 (m, 1H), 1.88–1.80 (m, 2H), 1.67–1.54 (m, 2H). HRMS [ESI], *m*/*z*: 434.1989 [M + H]^+^ for C_24_H_25_N_5_O_2_F. Anal. Calc. for C_24_H_24_N_5_O_2_F, %: C, 66.50; H, 5.58; N, 16.16; found, %: C, 66.58; H, 5.63; N, 16.24.

#### 3.1.2. Synthesis of Compounds **14**–**20**, and **28**

Compounds **14**–**20** were synthesized by following the procedure previously reported [13]. To a solution of 100 mg (0.23 mmol) of compound **III** trifluoroacetate salt (or compound **IV**) [12], dissolved in 5 mL of MeOH, were added acetone (1 mL), or cyclopentanone (0.30 mmol). After cooling (0 °C) Na(CN)BH_3_ (0.35 mmol) was added, and the mixture was stirred overnight at room temperature The reaction was quenched by adding a few drops of 5% aqueous KHSO_4_. After dilution with water, the mixture was corrected to pH 9 and extracted 3 times with 15 mL of methylene chloride (CH_2_Cl_2_). The collected organic phases were dried over anhydrous Na_2_SO_4_, filtered and concentrated under reduced pressure. The obtained crude products were purified by chromatography on silica gel, by eluting with a mixture of CH_2_Cl_2_/MeOH, 95/5 *v*/*v*, to achieve the desired final compounds in good yields. Compound **28** was obtained by following the same procedure, and by starting from 120 mg (0.2 mmol) of intermediate **V** [13].

*N-(3-(4-fluorobenzyloxy)phenyl)-1-isopropylpiperidine-4-carboxamide* (**14**) White solid, yield 85% (76 mg), M.p. 141–143 °C. IR cm^−1^: 3223, 2967, 2944, 2797, 1645, 1605, 1513, 1439, 1225, 1171, 1011, 838, 775. ^1^H NMR (300 MHz, CDCl_3_), *δ*_H_: 7.47 (s, 1H), 7.43–7.36 (m, 2H), 7.20 (t, 2H, *J* = 8.2 Hz), 7.07 (td, 2H, *J* = 10.8; 5.8 Hz), 6.92 (dd, 1H, *J* = 8.0; 1.2 Hz), 6.70 (dd, 1H, *J* = 7.9; 2.1 Hz), 5.02 (s, 2H), 3.00 (d, 2H, *J* = 11.9 Hz), 2.79 (heptet, 1H, *J* = 6.5 Hz), 2.35–2.20 (m, 3H), 2.06–1.94 (m, 2H), 1.93–1.78 (m, 2H), 1.07 (d, 6H, *J* = 6.5 Hz). HRMS [ESI], *m*/*z*: 371.2115 [M + H]^+^ for C_22_H_28_N_2_O_2_F. Anal. Calc. for C_22_H_27_N_2_O_2_F, %: C, 71.33; H, 7.35; N, 7.56; found, %: C, 71.42; H, 7.31; N, 7.55.

*N-(3-(4-fluorobenzyloxy)phenyl)-1-cyclopentylpiperidine-4-carboxamide* (**15**) Pale brown solid, yield 85% (78 mg), M.p. 183–185 °C. IR cm^−1^:3287, 2867, 2801, 2758, 1660, 1607, 1537, 1231, 1206, 1023, 830, 779. ^1^H NMR (300 MHz, DMSO-d_6_), *δ*_H_: 9.67 (s,1H), 7.48 (d, 2H, *J* = 9.0 -hz), 7.64–7.43 (m, 2H), 7.19 (d, 2H, *J* = 8.5 -Hz), 6.91 (d, 2H, *J* = 9.0 Hz), 5.02 (s, 2H), 3.00–2.70 (m, 3H), 2.35–2.10 (m, 2H), 1.80–1.60 (m, 4H). HRMS [ESI], *m*/*z*: 397.2291 [M + H]^+^ for C_24_H_30_FN_2_O_2_. Anal. Calc. for C_24_H_29_N_2_O_2_F, %: C, 72.70; H, 7.37; N, 7.07; found, %: C, 72.76; H, 7.43; N, 7.05.

*N-(4-(4-fluorobenzyloxy)phenyl)-1-isopropylpiperidine-4-carboxamide* (**16**) White solid, yield 75% (64 mg), M.p. 180–172 °C. IR cm^−1^:3304, 2920, 1652, 1602, 1527, 1243, 1228, 1009, 830. ^1^H NMR (300 MHz, DMSO-d_6_), *δ*_H_: 9.69 (s,1H), 7.48–7.45 (m, 4H), 7.19 (t, 2H, *J* = 8.5 -Hz), 6.91 (d, 2H, *J* = 9.0 Hz), 5.02 (s, 2H), 3.00–2.70 (m, 3H), 2.35–2.10 (m, 2H), 1.80–1.75 (m, 2H), 1.70–1.63 (m, 2H), 1.00 (d, 6H, *J* = 5.5). HRMS [ESI], *m*/*z*: 371.2137 [M + H]^+^ for C_22_H_28_FN_2_O_2_. Anal. Calc. for C_22_H_27_N_2_O_2_F, %: C, 71.33; H, 7.35; N, 7.56; found, %: C, 71.46; H, 7.44; N, 7.68.

*N-(4-(4-fluorobenzyloxy)phenyl)-1-cyclopentylpiperidine-4-carboxamide* (**17**) Pale brown solid, yield 50% (45 mg), M.p. 215–217 °C. IR cm^−1^: 3287, 2919, 1650, 1603, 1514, 1007, 1231, 828. ^1^H NMR (300 MHz, DMSO-d_6_), *δ*_H_: 9.65 (s, 1H), 7.50 (d, 2H, *J* = 9.0 Hz), 7.65–7.41 (m, 2H), 7.18 (d, 2H, *J* = 8.5 Hz), 6.89 (d, 2H, *J* = 9.0), 4.98 (s, 2H), 3.30–3.15 (m, 2H), 3.00–2.80 (m, 3H), 2.30–2.10 (m, 1H), 2.05–1.90 (m, 4H), 1.80–1.55 (m, 8H). HRMS [ESI], *m*/*z*: 397.2287 [M + H]^+^ for C_24_H_30_FN_2_O_2_. Anal. Calc. for C_24_H_29_N_2_O_2_F, %: C, 72.70; H, 7.37; N, 7.07; found, %: C, 72.81; H, 7.44; N, 7.12.

*N-(2-(4-fluorobenzyloxy)phenyl)-1-isopropylpiperidine-4-carboxamide* (**18**) Pink solid, yield 69% (58 mg), M.p. 180–182 °C. IR cm^−1^: 3282, 1732, 1662, 1611, 1589, 1536, 1374, 1263, 1107, 852, 781. ^1^H NMR (300 MHz, DMSO-d_6_), *δ*_H_: 9.00 (s, 1H), 7.75 (d, 1H, *J* = 7.5), 7.51 (t, 2H, *J* = 7.5), 7.20 (t, 2H, *J* = 9.0), 7.10–7.00 (m, 2H), 6.88 (t, 1H, *J* = 8.0), 5.13 (s, 2H), 3.00–2.70 (m, 3H), 2.35–2.10 (m, 3H), 1.80–1.60 (m, 4H), 1.00 (d, 6H, *J* = 6.0). HRMS [ESI], *m*/*z*: 371.2134 [M + H]^+^ for C_22_H_28_FN_2_O_2_. Anal. Calc. for C_22_H_27_N_2_O_2_F, %: C, 71.33; H, 7.35; N, 7.56; found, %: C, 71.54; H, 7.51; N, 7.48.

*N-(2-(4-fluorobenzyloxy)phenyl)-1-cyclopentylpiperidine-4-carboxamide* (**19**) White solid, yield 65% (60 mg), M.p. 183–185 °C. IR cm^−1^: 3284, 1654, 1603, 1537, 1371, 1224, 1048, 744. ^1^H NMR (300 MHz, DMSO-d_6_), *δ*_H_: 8.96 (s, 1H), 7.76 (d, 1H, *J* = 7.5), 7.60–7.45 (m, 2H), 7.21 (d, 2H, *J* = 9.0), 7.10–7.00 (m, 2H), 6.89 (t, 1H, *J* = 8.0), 5.11 (s, 2H), 3.10–3.00 (m, 3H), 3.00–2.80 (m, 2H), 2.30–2.10 (m, 1H), 2.05–1.90 (m, 4H), 1.80–1.50 (m, 8H). HRMS [ESI], *m*/*z*: 397.2294 [M + H]^+^ for C_24_H_30_FN_2_O_2_. Anal. Calc. for C_24_H_29_N_2_O_2_F, %: C, 72.70; H, 7.37; N, 7.07; found, %: C, 72.71; H, 7.39; N, 7.06.

*N-(2-(4-chlorobenzyloxy)phenyl)-1-isopropylpiperidine-4-carboxamide* (**20**) White solid, yield 87% (98 mg), M.p. 208–210 °C. IR cm^−1^: 3499, 3395, 3362, 2668, 1668, 1537, 1452, 1200, 998, 751. ^1^H NMR (300 MHz, DMSO-d_6_), *δ*_H_: 10.05 (s, 1H), 9.20 (s, 1H), 7.72 (d, 1H, *J* = 7.8 Hz), 7.51 (d, 2H, *J* = 8.5 Hz), 7.45 (d, 2H, *J* = 8.5 Hz), 7.05 (d, 2H, *J* = 4.5 Hz), 6.96–6.85 (m, 1H), 5.16 (s, 2H), 3.41 (d, 2H, *J* = 11.6 Hz), 3.00–2.79 (m, 2H), 2.78–2.64 (m, 1H), 2.10–1.92 (m, 4H), 1.26 (d, 6H, *J* = 6.5 Hz). HRMS [ESI], *m*/*z*: 387.1832 [M + H]^+^ for C_22_H_28_N_2_O_2_Cl. Anal. Calc. for C_22_H_27_N_2_O_2_Cl, %: C, 68.29; H, 7.03; N, 7.24; found, %: C, 68.38; H, 7.12; N, 7.22.

*N-(2-(3-chlorobenzyloxy)phenyl)-1-isopropylpiperidine-4-carboxamide* (**28**) White solid, yield 87% (158 mg), M.p. 211–213 °C. IR cm^−1^: 3499, 3395, 3362, 2668, 1668, 1537, 1452, 1200, 998, 800. ^1^H NMR (300 MHz, DMSO-d_6_), *δ*_H_: 9.82 (s, 1H), 7.98 (s, 1H), 7.90–7.81 (m, 1H), 7.60–7.53 (m, 2H), 7.44 (s, 1H), 7.28 (s, 1H), 7.22 (t, 1H, *J* = 7.8 Hz), 7.18–7.11 (m, 1H), 6.71 (d, 1H, *J* =7.6 Hz), 5.19 (s, 2H), 2.80 (d, 2H, *J* = 10.6 Hz), 2.64 (septet, 1H, *J* = 6.3 Hz), 2.30–2.17 (m, 1H), 2.30–2.17 (m, 1H), 2.07 (t, 2H, *J* = 10.9 Hz), 1.78–1.66 (m, 2H), 1.65–1.46 (m, 2H), 0.94 (d, 6H, *J* = 6.4 Hz). HRMS [ESI], *m*/*z*: 387.1832 [M + H]^+^ for C_22_H_28_N_2_O_2_Cl. Anal. Calc. for C_22_H_27_N_2_O_2_Cl, %: C, 68.29; H, 7.03; N, 7.24; found, %: C, 68.38; H, 7.12; N, 7.22.

#### 3.1.3. Synthesis of *N*-(2-(4-Chlorobenzyloxy)phenyl)-1-amidinopiperidine-4-carboxamide hydrochloride (**21**)

1,3-bis(tertbutoxycarbonyl)-2-methyl-2-thiopseudourea (80 mg, 0.31 mmol), mercury (II) chloride HgCl_2_ (87 mg, 0.31 mmol) and triethylamine (0.11 mL, 0.78 mmol) were added to a cooled solution (0 °C) of compound **IV** (100 mg, 0.26 mmol) in 5ml of dry DMF. The reaction mixture was stirred for 1 h at 0 °C and 24 h at room temperature. After dilution with 10 mL of ethyl acetate the mixture stirring was continued for 15 min. The precipitate was filtered off and washed 3 times with 5 mL of EtOAc. The combined organic phases were washed with brine, dried over Na_2_SO_4_, and concentrated under reduced pressure. The obtained residue was purified by silica gel chromatography, by eluting with petroleum ether/EtOAc 90:10, *v*/*v*, to afford the *N,N*′-bis-BOC protected derivative, which was finally treated with HCl gas in chloroform solution to afford the HCl salt of the desired product. Dark grey oil, yield 76% (72 mg), IR cm^−1^: 3350, 3212, 2875, 1651, 1603, 1453, 1401, 1015, 756. ^1^H NMR (300 MHz, DMSO-d_6_), δ_H_: 8.93 (s, 1H), 7.77 (d, 1H, *J* = 7.8 Hz), 7.52 (d, 2H, *J* = 8.5 Hz), 7.41 (d, 2H, *J* = 8.5 Hz), 7.02.6.99 (m, 2H), 6.92–6.82 (m, 1H), 5.16 (s, 2H), 2.93 (d, 1H, *J* = 12.1 Hz), 0.78–2.70 M, 1H), 2.48 (s, 2H), 1.64 (d, 1H, *J* = 12.4 Hz), 1.45 (qd, 1H, *J* = 12.2; 4.0 Hz). HRMS [ESI], *m*/*z*: 387.1585 [M + H]^+^ for C_20_H_24_N_4_O_2_Cl. Anal. Calc. for C_20_H_23_N_4_O_2_Cl %: C, 62.09; H, 5.99; N, 14.48; found, %: C, 62.18; H, 6.05; N, 14.46.

### 3.2. Enzymatic Assays

#### 3.2.1. Cholinesterases Inhibition Assay

The test compounds for their inhibitory activity toward AChE (from electric eel) and BChE (from horse serum) as well were evaluated by applying the Ellman’s assay with some modifications [24]. The anti-AChE activity was determined in a reaction mixture containing 20 μL of a solution of AChE (0.9 U/mL in 0.1 M pH 8.0 phosphate buffer, PB), 20 μL of a solution of 5,5-dithio-bis-(2-nitrobenzoic) acid (DTNB 3.3 mM in 0.1 M pH 7.0 PB, containing 0.1 mM NaHCO_3_), 20 μL of a solution of the test compound (five to seven concentrations, ranging from 1 × 10^−4^ to 1 × 10^−10^ M in 0.1 M pH 8.0 PB), and 120 μL of pH 8.0 PB. After incubation for 20 min at 25 °C, acetylthiocholine iodide (20 μL of 0.05 mM water solution) was added as the substrate, the hydrolysis rates of the substrate monitored at 412 nm for 5.0 min at 25 °C, and the initial reaction rate was determined within 60 s. The concentration of the inhibitor required to diminish by 50% the rate of the control (IC_50_), was calculated by nonlinear (sigmoid) regression of the response/concentration (log) curve, by using Prisma GraphPad software (vers. 5.01). From three independent IC_50_ values, the inhibition constants K_i_ were calculated by using the Cheng−Prousoff equation [15]. Relative SEM values (standard error of means) were all found less than 5% of the means. BChE inhibitory activity was determined similarly, by using a solution of BChE (1.8 U/mL in 0.1 M pH 8.0 PB), and butyrylthiocholine iodide (0.05 mM) as substrate. To determine the type of inhibition for the most potent AChE and BChE inhibitors, the Lineweaver–Burk eq (1/v vs. 1/[S]) was fitted for varying concentrations of substrates (25–300 μM) in the absence or presence of inhibitor at least four different concentrations and by using fixed amounts of enzymes. Replotting the slopes of the above plots against the inhibitor concentration yielded the K_i_ value as the *x*-axis intercept.

#### 3.2.2. Coagulative Serine Proteases Thrombin and fXa Inhibition Assay

The test compounds were evaluated in vitro for their inhibitory activity toward fXa and fIIa, determining the hydrolysis rates of the synthetic chromogenic substrates, monitored at 405 nm [13]. Enzymes and substrates were used as follows, in final concentrations: 2 nM human fXa and 0.04 mM S-2765 (Z-D-Arg-Gly-Arg-p-NA) from Chromogenix AB-Instrumentation Laboratories (Milan, Italy), 0.41 U·mL^−1^ bovine thrombin from Sigma-Aldrich (Milan, Italy), and 0.05 mM S-2238 (D-Phe-Pip-Arg-p-NA) from Chromogenix AB-Instrumentation Laboratories (Milan, Italy). Enzyme solutions were incubated with DMSO solutions of the test inhibitor (DMSO did not exceed 1%) in various concentrations (0.001−50 μM) before the respective chromogenic substrates were added to start the enzyme kinetics. The kinetic studies were performed at pH 8. Reactions were initiated by adding 100 μL of substrate solutions and monitoring the increase in absorbance for 5 min. The initial velocities were determined within 60 s and the concentration of the inhibitor required to diminish by 50% the control velocity (IC_50_), calculated by nonlinear (sigmoid) regression. Three independent IC_50_ values were determined for calculating inhibition constants K_i_ using the Cheng−Prousoff equation [15,28].

### 3.3. Chemoinformatics and Computational Chemistry

The Multi-fingerprint Similarity Search aLgorithm (MuSSeL) is released as a ligand-based predictive web server to find putative protein drug targets of new conceived small molecules or to repurpose existing bioactive compounds. Predictions are computed by screening a collection, including 611,333 small molecules provided with high-quality experimental bioactivity data covering 3357 protein drug targets, which were rationally selected from the latest release of ChEMBLdb (version 25 March 2019).

### 3.4. Molecular Modelling

Crystal structures of AChE, BChE, factor Xa, and thrombin were retrieved from the Protein Data Bank (PDB) by selecting entries 1EVE, 6SAM, 3K9X and 1UVS, respectively [34,35,36,37]. Protein structures were geometrically optimized and energetically minimized by employing the protein preparation tool available in the Schrodinger Suite [38,39]. Moreover, ligands were treated by using Ligprep Tool [40], allowing us to evaluate all possible tautomers and protonation states at physiological pH. Molecular docking simulations were performed by using GLIDE software [41]. All the enclosing boxes were centered on the corresponding cognate ligands. For the sake of completeness, to corroborate the validity of docking protocols, redocking analyses were carried out on the cognate ligands in their binding pockets. Satisfactorily, the root mean square deviation (RMSD) values between original and best docked poses were equal to 0.423, 0.349, 0.988 and 0.752 Å for AChE, BChE, factor Xa and thrombin, respectively.

Furthermore, a more detailed study was performed on the top-scored docking solutions of **1** vs. AChE, **1** vs. thrombin, **22** vs. BChe and **22** vs. FXa. The Monte Carlo sampling method implemented in Prime was employed [42,43] to explore different positions and conformations of ligands within the binding pockets. Specifically, VSGB 2.0 solvation model [44], and the OPLS3 force field [45] were used. The dielectric constant of the solvent was equal to 80, thus assuming water as medium. A region of 6 Å from ligands was selected for refinement and 100 steps of Monte Carlo minimization were set as default options.

BioGPS software [46] was used to energetically feature AChE, BChE, factorXa and thrombin binding pockets. CRY, H, N1 and O GRID probes, which indicate hydrophobic, shape, hydrogen bond donor and hydrogen bond acceptor interactions, respectively, were computed and pocket comparisons (i.e., AChE vs. thrombin and BChE vs. factor Xa) were made based on molecular interaction fields (MIFs).

## 4. Conclusions

In reviewing the therapeutic approach to AD, a role has been proposed for direct oral anticoagulants (DOACs). These molecules (e.g., dabigatran, rivaroxaban, apixaban) may counteract the cerebral amyloid angiopathy (CAA), that a pathological alteration in cerebral blood vessels induced by Aβ deposits [47]. Ultimately, DOACs may help to decrease vascular-driven progression in AD neurodegenerative and cognitive changes.

In this study, using computational tools of virtual screening and molecular docking simulations, we investigated an in-house developed library of thirty previously and newly synthesized isonipecotamide-based inhibitors of blood coagulation factors, namely thr and/or fXa, the purpose being the discovery of new multimodal agents inhibiting other AD-related enzymes, such as AChE and BChE, while maintaining almost intact their anticoagulant potency. Cross-docking screening shed light into the structure-activity relationships, whereas molecular interaction field (MIFs) calculations helped the identification of common features between the inhibitors’ binding sites of the investigated AD-related target enzymes, thus highlighting their modeling utility in drug design and hit-to-lead optimization of new anti-AD small molecule candidates.

As a major outcome, two novel DOAC hits, namely the 1-(pyridin-4-yl)- (**1**) and 1-amidino- (**23**) isonipecotanilide derivatives, which proved to inhibit contemporarily thr/AChE and fXa/BChE, respectively, with inhibition constants in the low nanomolar range, deserve investigation in further ex vivo and in vivo animal models of AD and related cognitive impairments.

## Figures and Tables

**Table 1:**
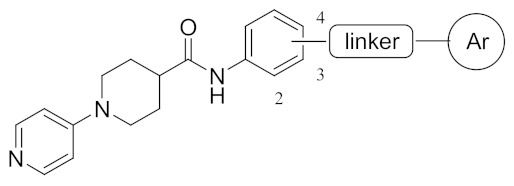
Inhibitory activity of analogs of the isonipecotanilide **1** on blood coagulation factors (bovine thr, human recombinant fXa) and cholinesterases (*ee*AChE, *eq*BChE).

				K_i_ (μM) ^1^			
Cmpd	Linker pos.	Linker	F-pos.	thr ^2^	fXa ^3^	AChE ^4^	BChE ^5^
**1 ^6^**	3	OCH_2_	3′	0.006	5.60	0.058	6.95
**2 ^6^**	3	OCH_2_	4′	0.210	1.10	0.240	1.21
**3 ^6^**	3	CH_2_O	4′	26.8	0.774	0.165	2.60
**4 ^6^**	4	OCH_2_	3′	6.70	25.1	3.88	0.425
**5 ^6^**	2	OCH_2_	3′	(10 ± 5%)	(2 ± 2%)	(12 ± 8%)	0.655
**6 ^6^**	3	OCH_2_CH_2_	3′	2.14	0.488	0.369	1.75
**7 ^6^**	3	OCH_2_CH_2_	4′	4.46	0.133	0.650	5.63
**8 ^6^**	3	CH_2_CH_2_	4′	3.72	37.7	0.495	8.20
**9**	3	CONH	4′	4.47	4.04	0.270	8.25
**10**	3	CONHCH_2_	4′	10.6	2.40	0.255	0.370
**11**	3	SO_2_NHCH_2_	4′	11.2	3.09	0.155	4.10
**12**	3	NHCONH	4′	26.7	16.5	0.735	(15 ± 7%)
dabigatran				0.0042	5.10		(36 ± 7%)
apixaban					0.00012		
donepezil						0.021	2.25

^1^ K_i_ values determined by applying the Cheng–Prousoff equation to IC_50_ values, by regression (GraphPad Prism software ver. 5.01), or inhibition percentage ± SEM at 50 μM (thr and fXa) or 10 μM (AChE and BChE). Data are means of three independent measurements, each one performed in triplicate (SEM < 5%). Dabigatran and apixaban as positive controls against blood coagulation factors; donepezil as positive control against cholinesterases. ^2^ Bovine serum thrombin. ^3^ Human recombinant fXa. ^4^ Electric eel acetylcholinesterase. ^5^ Horse serum butyrylcholinesterase. ^6^ Previously reported [15].

**Table 2:**
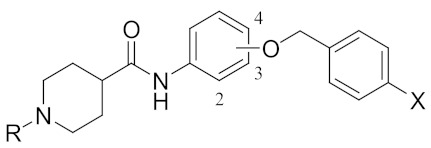
Inhibition of *N*1-substituted isonipecotanilide derivatives on blood coagulation factors (thr, fXa) and cholinesterases (AChE, BChE).

				K_i_ (μM) ^1^			
Compd	OCH_2_ pos.	R	X	thr ^2^	fXa ^3^	AChE ^4^	BChE ^5^
**13**	3	C(=NH)NH_2_	F	20.0	33.0	10.0	0.260
**14**	3	*i*-Pr	F	(5 ± 5%)	(28 ± 8%)	15.5	0.130
**15**	3	*c*-Pent	F	30.0	(40 ± 6%)	(27 ± 9%)	0.115
**16**	4	*i*-Pr	F	(11 ± 6%)	(5 ± 5%)	6.25	2.36
**17**	4	*c*-Pent	F	(25 ± 6%)	(28 ± 9%)	3.52	0.255
**18**	2	*i*-Pr	F	5.35	10.5	(15 ± 5%)	0.378
**19**	2	*c*-Pent	F	(12 ± 3%)	(28 ± 3%)	(35 ± 7%)	0.215
**20**	2	*i*-Pr	Cl	(20 ± 5%)	0.484	(11 ± 2%)	0.105
**21**	2	C(=NH)NH_2_	Cl	25.9	60.6	(38 ± 3%)	8.20

^1–5^. See footnotes in Table 1.

**Table 3:**
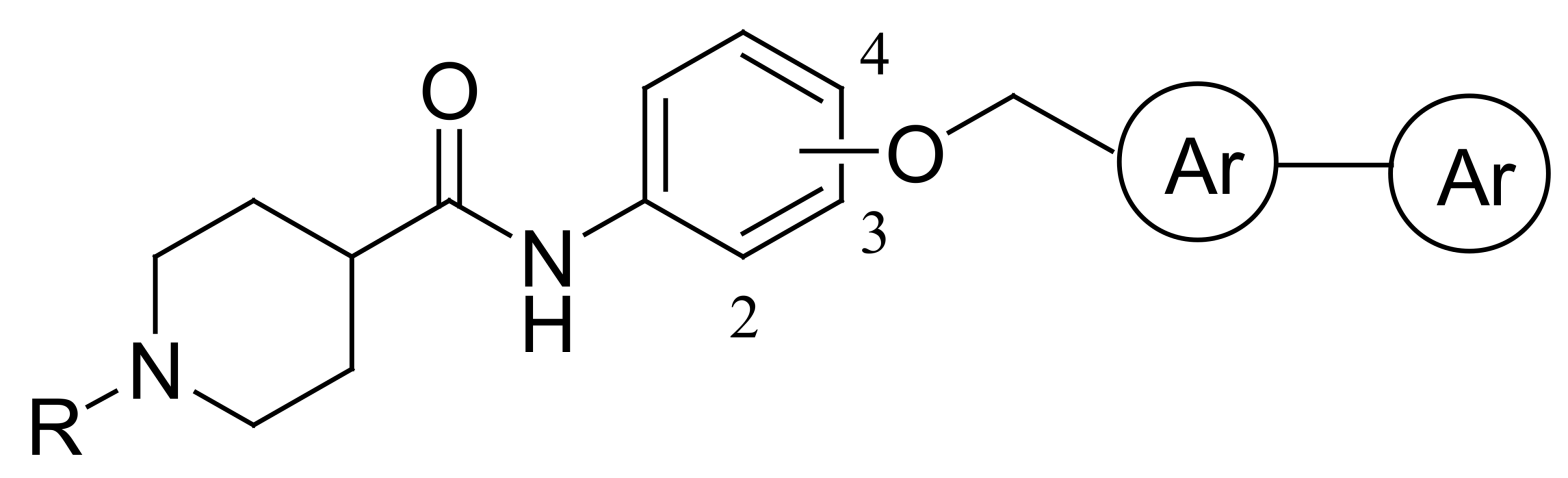
Anticholinesterase activity of isonipecotanilide-based fXa selective inhibitors.

	OCH_2_			K_i_ (μM) ^1^			
Compd	Pos	R	Ar–Ar	thr ^2^	fXa ^3^	AChE ^4^	BChE ^5^
**22**	3	*i*-Pr	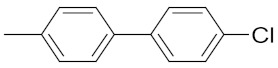	(5 ± 2%)	0.165	3.33	(35 ± 10%)
**23**	3	C(=NH)NH_2_		2.04	0.031	5.80	0.090
**24**	3	*i*-Pr	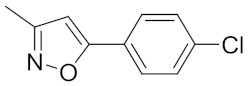	(15 ± 5%)	0.218	6.30	3.06
**25**	3	C(=NH)NH_2_		0.630	0.026	11.0	7.67
**26**	2	*i*-Pr		13.7	0.007	11.5	1.11
**27**	2	C(=NH)NH_2_		21.2	0.060	(16 ± 3%)	10.0
**28**	3	*i*-Pr	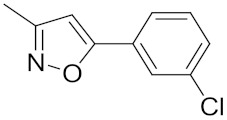	23.0	0.867	7.00	4.10
**29**	3	C(=NH)NH_2_		15.3	0.388	10.0	1.12
**30**	3	*i*-Pr	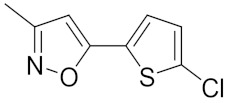	16.0	0.090	5.10	2.13
**31**	3	C(=NH)NH_2_		15.0	0.015	(31 ± 6%)	1.62

^1–5^ See footnotes in Table 1.

## Data Availability

All data presented in this study are available in the article and in Supplementary Informations.

## Supplementary Materials

The following materials are available online. MuSSeL prediction output as pdf attached file; Figures S1 and S2, inhibition mechanism profiles; Tables S1 and S2, Similarity matrices of MIFs (BioGPS); ^1^H NMR spectra of the newly synthesized compounds.

## Sample Availability

Samples of the compounds presented are available from the authors.

## References

[1] Patterson C. (2018). World Alzheimer Report 2018: The State of the Art of Dementia Research: New Frontiers. https://www.alz.co.uk/research/.

[2] Knopman D.S., Amieva H., Petersen R.C., Chételat G., Holtzman D.M., Hyman B.T., Nixon R.A., Jones D.T. (2021). Alzheimer disease. Nat. Rev. Dis. Primers.

[3] Kumar A., Singh A., Ekavali A. (2015). A review on Alzheimer’s disease pathophysiology and its management: An update. Pharmacol. Rep..

[4] Geldenhuys W.J., Darvesh A.S. (2015). Pharmacotherapy of Alzheimer’s disease: Current and future trends. Expert Rev. Neurother..

[5] Zupanica E., Krambergera M.G., von Eulerd M., Norrvingf B., Winblada B., Secniki J., Fastbomh J., Eriksdotterg M., Garcia-Ptaceki S. (2020). Secondary stroke prevention after ischemic stroke in patients with Alzheimer’s disease and other dementia disorders. J. Alzheimer’s Dis..

[6] Scheffer S., Hermkens D.M.A., van der Weerd L., de Vries H.E., Daemen M.J.A.P. (2021). Vascular hypothesis of Alzheimer disease. Arterioscler. Thromb. Vasc. Biol..

[7] Grossmann K. (2021). Alzheimer’s disease—Rationales for potential treatment withthe thrombin inhibitor dabigatran. Int. J. Mol. Sci..

[8] Marangoni M.N., Braun D., Situ A., Moyano A.L., Kalinin S., Polak P., Givogri M.I., Feinstein D.L. (2016). Differential effects on glial activation by a direct versus an indirect thrombin inhibitor. J. Neuroimmunol..

[9] Iannucci J., Renehan W., Grammas P. (2020). Thrombin, a mediator of coagulation, inflammation, and neurotoxicity at the neurovascular interface: Implications for Alzheimer’s disease. Front. Neurosci..

[10] Cortes-Canteli M., Kruyer A., Fernandez-Nueda I., Marcos-Diaz A., Ceron C., Richards A.T., Jno-Charles O.C., Rodriguez I., Callejas S., Norris E. (2019). Long-term dabigatran treatment delays Alzheimer’s disease pathogenesis in the TgCRND8 mouse model. J. Am. Coll. Cardiol..

[11] Ihara M., Saito S. (2020). Drug repositioning for alzheimer’sdisease: Finding hidden clues in old drugs. J. Alzheimer’s Dis..

[12] de Candia M., Liantonio F., Carotti A., De Cristofaro R., Altomare C. (2009). Fluorinated benzyloxyphenyl piperidine-4-carboxamides with dual function against thrombosis: Inhibitors of factor Xa and platelet aggregation. J. Med. Chem..

[13] Lopopolo G., Fiorella F., de Candia M., Nicolotti O., Martel S., Carrupt P.A., Altomare C. (2011). Biarylmethoxy isonipecotanilides as potent and selective inhibitors of blood coagulation factor Xa. Eur. J. Pharm. Sci..

[14] Lopopolo G., de Candia M., Panza L., Romano M.R., Lograno M.D., Campagna F., Altomare C. (2012). β-D-glucosyl conjugates of highly potent inhibitors of blood coagulation factor Xa bearing 2-chorothiophene as a P1 motif. ChemMedChem.

[15] de Candia M., Fiorella F., Lopopolo G., Carotti A., Romano M.R., Lograno M.D., Martel S., Carrupt P.A., Belviso B.D., Caliandro R. (2013). Synthesis and biological evaluation of direct thrombin inhibitors bearing 4-(piperidin-1-yl)pyridine at the P1 position with potent anticoagulant activity. J. Med. Chem..

[16] Belviso B.D., Caliandro R., de Candia M., Zaetta G., Lopopolo G., Incampo F., Colucci M., Altomare C. (2014). How a β-D-glucoside side chain enhances binding affinity to thrombin of inhibitors bearing 2-chlorothiophene as P1 moiety: Crystallography, fragment deconstruction study, and evaluation of antithrombotic properties. J. Med. Chem..

[17] Alberga D., Trisciuzzi D., Montaruli M., Leonetti F., Mangiatordi G.F., Nicolotti O. (2019). A new approach for drug target and bioactivity prediction: The Multi-fingerprint Similarity Search aLgorithm (MuSSeL). J. Chem. Inf. Model..

[18] Montaruli M., Alberga D., Ciriaco F., Trisciuzzi D., Tondo A.R., Mangiatordi G.F., Nicolotti O. (2019). Accelerating drug discovery by early protein drug target prediction based on multi-fingerprint similarity search. Molecules.

[19] Cavalli A., Bolognesi M.L., Minarini A., Rosini M., Tumiatti V., Recanatini M., Melchiorre C. (2008). Multi-target-directed ligands to combat neurodegenerative diseases. J. Med. Chem..

[20] Maramai S., Benchekroun M., Gabr M.T., Yahiaoui S. (2020). Multitarget therapeutic strategies for Alzheimer’s disease: Review on emerging target combinations. BioMed Res. Int..

[21] De Freitas Silva M., Dias K.S.T., Gontijo V.S., Ortiz C.J.C., Viegas C. (2018). Multi-target directed drugs as a modern approach for drug design towards Alzheimer’s disease: An update. Curr. Med. Chem..

[22] Mezeiova E., Chalupova K., Nepovimova E., Gorecki L., Prchal L., Malinak D., Kuca K., Soukup O., Korabecny J. (2019). Donepezil derivatives targeting amyloid-β cascade in Alzheimer’s disease. Curr. Alzheimer Res..

[23] Ha Z.Y., Mathew S., Yeong K.Y. (2020). Butyrylcholinesterase: A multifaced pharmacological target and too. Curr. Prot. Pept. Sci..

[24] de Candia M., Zaetta G., Denora N., Tricarico D., Majellaro M., Cellamare S., Altomare C.D. (2017). New azepino[4,3-*b*]indole derivatives as nanomolar selective inhibitors of human butyrylcholinesterase showing protective effects against NMDA-induced neurotoxicity. Eur. J. Med. Chem..

[25] Nicolotti O., Giangreco I., Miscioscia T.F., Carotti A. (2009). Improving quantitative structure-activity relationships through multiobjective optimization. J. Chem. Inf. Model..

[26] Nicolotti O., Miscioscia T.F., Carotti A., Leonetti F., Carotti A. (2008). An integrated approach to ligand- and structure-based drug design: Development and application to a series of serine protease inhibitors. J. Chem. Inf. Model..

[27] Purgatorio R., de Candia M., Catto M., Carrieri A., Pisani L., De Palma A., Toma M., Ivanova O.A., Voskressensky L.G., Altomare C.D. (2019). Investigating 1,2,3,4,5,6-hexahydroazepino[4,3-*b*]indole as scaffold of butyrylcholinesterase-selective inhibitors with additional neuroprotective activities for Alzheimer’s disease. Eur. J. Med. Chem..

[28] Cheng Y., Prusoff W.H. (1973). Relation between the inhibition constant (K_i_) and the concentration of inhibitor which causes fifty per cent inhibition (IC_50_) of an enzymic reaction. Biochem. Pharmacol..

[29] Gao X.H., Liu L.B., Liu H.R., Tang J.J., Kang L., Wu H., Cui P., Yan J. (2017). Structure–activity relationship investigation of benzamide and picolinamide derivatives containing dimethylamine side chain as acetylcholinesterase inhibitors. J. Enzyme Inhib. Med. Chem..

[30] Clark D.E., Pickett S.D. (2000). Computational methods for the prediction of “drug-likeness”. Drug Discov. Today.

[31] Geldenhuys W.J., Mohammad A.S., Adkins C.E., Lockman P.R. (2015). Molecular determinants of blood-barin-barrier permeation. Ther. Deliv..

[32] Cheng F., Li W., Zhou Y., Shen J., Wu Z., Liu G., Lee P.W., Tang Y.J. (2012). admetSAR: A comprehensive source and free tool for assessment of chemical ADMET properties. Chem. Inf. Model..

[33] Nazaré M., Will D.W., Matter H., Schreuder H., Ritter K., Urmann M., Essrich M., Bauer A., Wagner M., Czech J. (2005). Probing the subpockets of factor Xa reveals two binding modes for inhibitors based on a 2-carboxyindole scaffold: A study combining structure–activity relationship and X-ray crystallography. J. Med. Chem..

[34] Kryger G., Silman I., Sussman J.L. (1999). Structure of acetylcholinesterase complexed with E2020 (Aricept^®^): Implications for the design of new anti-Alzheimer drugs. Structure.

[35] Košak U., Strašek N., Knez D., Jukič M., Žakelj S., Zahirović A., Pišlar A., Brazzolotto X., Nachon F., Kos J. (2020). N-Alkylpiperidine carbamates as potential anti-Alzheimer’s agents. Eur. J. Med. Chem..

[36] Shi Y., Li C., O’Connor S.P., Zhang J., Shi M., Bisaha S.N., Wang Y., Sitkoff D., Pudzianowski A.T., Huang C. (2009). Aroylguanidine-based factor Xa inhibitors: The discovery of BMS-344577. Bioorg. Med. Chem. Lett..

[37] Engh R.A., Brandstetter H., Sucher G., Eichinger A., Baumann U., Bode W., Huber R., Poll T., Rudolph R., von der Saal W. (1996). Enzyme flexibility, solvent and ‘weak’ interactions characterize thrombin–ligand interactions: Implications for drug design. Structure.

[38] Madhavi Sastry G., Adzhigirey M., Day T., Annabhimoju R., Sherman W. (2013). Protein and ligand preparation: Parameters, protocols, and influence on virtual screening enrichments. J. Comput. Aided Mol. Des..

[39] Schrödinger Release (2016). Protein Preparation Wizard.

[40] Schrödinger Release (2020). LigPrep.

[41] Friesner R.A., Banks J.L., Murphy R.B., Halgren T.A., Klicic J.J., Mainz D.T., Repasky M.P., Knoll E.H., Shelley M., Perry J.K. (2004). Glide: A new approach for rapid, accurate docking and scoring. 1. Method and assessment of docking accuracy. J. Med. Chem..

[42] Schrödinger Release (2020). Prime.

[43] Jacobson M.P., Pincus D.L., Rapp C.S., Day T.J.F., Honig B., Shaw D.E., Friesner R.A. (2004). A hierarchical approach to all-atom protein loop prediction. Proteins Struct. Funct. Bioinform..

[44] Li J., Abel R., Zhu K., Cao Y., Zhao S., Friesner R.A. (2011). The VSGB 2.0 model: A next generation energy model for high resolution protein structure modeling: The VSGB 2.0 energy model. Proteins.

[45] Harder E., Damm W., Maple J., Wu C., Reboul M., Xiang J.Y., Wang L., Lupyan D., Dahlgren M.K., Knight J.L. (2016). OPLS3: A force field providing broad coverage of drug-like small molecules and proteins. J. Chem. Theory Comput..

[46] Siragusa L., Cross S., Baroni M., Goracci L., Cruciani G. (2015). BioGPS: Navigating biological space to predict polypharmacology, off-targeting, and selectivity. Proteins Struct. Funct. Bioinform..

[47] Grossmann K. (2020). Anticoagulants for treatment of Alzheimer’s disease. J. Alzheimer’s Dis..

