# Supplementary material for: First-in-Class Isonipecotamide-Based Thrombin and Cholinesterase Dual Inhibitors with Potential for Alzheimer Disease
Source: Molecules. 2021 Aug 27;26(17):5208. doi: 10.3390/molecules26175208 (PMC8434007; doi:10.3390/molecules26175208)

First-in-class isonipecotamide-based thrombin and cholinesterase dual inhibitors with potential for Alzheimer disease

Rosa Purgatorio,¶ Nicola Gambacorta,¶ Modesto de Candia,* Marco Catto, Mariagrazia Rullo, Leonardo Pisani, Orazio Nicolotti and Cosimo D. Altomare

[1] Department of Pharmacy – Pharmaceutical Sciences, University of Bari "Aldo Moro", via Orabona 4, I-70125, Bari (Italy)
* Correspondence: modesto.decandia@uniba.it

Supplementary Information

1. **MuSSeL prediction output** **pag. 2**
2. **Inhibition mechanism profiles** **pag. 6**
3. **Molecular modelling** **pag. 8**
4. **1H NMR spectra** **pag. 9**

1. EMuSSeL prediction output

Prediction

Prediction for O=C(Nc2cccc(OCc1cccc(F)c1)c2)C4CCN(c3ccncc3)CC4

Position	Target	pIC50	τ
1	Anandamide amidohydrolase:Homo sapiens	7.04 ± 2.19	0.209
2	Platelet-derived growth factor receptor beta:Homo sapiens	6.68 ± 2.21	0.205
3	Anandamide amidohydrolase:Rattus norvegicus	6.65 ± 2.27	0.194
4	Sphingosine 1-phosphate receptor Edg-1:Homo sapiens	7.60 ± 2.28	0.192
5	C-C chemokine receptor type 3:Homo sapiens	7.40 ± 2.31	0.187
6	Cytochrome P450 2D6:Homo sapiens	5.44 ± 2.35	0.181
7	Neuropeptide Y receptor type 5:Homo sapiens	7.69 ± 2.35	0.180
8	Caspase-3:Homo sapiens	6.12 ± 2.36	0.180
9	Caspase-7:Homo sapiens	6.87 ± 2.36	0.180
10	Tyrosine-protein kinase JAK3:Homo sapiens	7.16 ± 2.36	0.179
11	Platelet activating factor receptor:Homo sapiens	6.94 ± 2.37	0.179
12	MAP kinase ERK2:Mus musculus	7.33 ± 2.37	0.178
13	Leukotriene A4 hydrolase:Homo sapiens	6.97 ± 2.37	0.178
14	Hepatocyte growth factor receptor:Homo sapiens	7.23 ± 2.38	0.177
15	Neuronal acetylcholine receptor protein alpha-7 subunit:Rattus norvegicus	6.50 ± 2.38	0.177
16	Cytochrome P450 3A4:Homo sapiens	5.50 ± 2.38	0.177
17	Muscarinic acetylcholine receptor M4:Homo sapiens	6.14 ± 2.38	0.176
18	Poly [ADP-ribose] polymerase-1:Homo sapiens	7.11 ± 2.38	0.176
19	Glycine transporter 2:Homo sapiens	6.31 ± 2.39	0.176
20	Melanin-concentrating hormone receptor 1:Homo sapiens	7.18 ± 2.39	0.175
21	Epoxide hydratase:Homo sapiens	7.45 ± 2.39	0.175
22	Tyrosine-protein kinase JAK2:Homo sapiens	7.37 ± 2.39	0.175
23	Intestinal alkaline phosphatase:Mus musculus	5.03 ± 2.39	0.175
24	PRMT5/MEP50 complex:Homo sapiens	6.42 ± 2.39	0.175
25	cAMP and cAMP-inhibited cGMP 3',5'-cyclic phosphodiesterase 10A:Rattus norvegicus	7.37 ± 2.40	0.174

Position	Target	pIC50	τ
26	Muscarinic acetylcholine receptor M3:Homo sapiens	7.47 ± 2.40	0.173
27	Cytochrome P450 2C9:Homo sapiens	5.37 ± 2.40	0.173
28	Acetylcholinesterase:Homo sapiens	6.27 ± 2.41	0.173
29	Tyrosine-protein kinase JAK1:Homo sapiens	8.08 ± 2.41	0.173
30	MAP kinase ERK2:Homo sapiens	8.10 ± 2.41	0.172
31	Cholecystokinin A receptor:Cavia porcellus	6.48 ± 2.41	0.172
32	Vascular endothelial growth factor receptor 2:Homo sapiens	6.88 ± 2.41	0.172
33	Ceramide glucosyltransferase:Homo sapiens	7.39 ± 2.42	0.171
34	Arachidonate 5-lipoxygenase:Rattus norvegicus	5.94 ± 2.42	0.171
35	MAP kinase p38 alpha:Homo sapiens	7.07 ± 2.42	0.171
36	Matrix metalloproteinase 9:Homo sapiens	7.23 ± 2.42	0.171
37	Calpain 2:Sus scrofa	5.11 ± 2.42	0.171
38	Vanilloid receptor:Homo sapiens	6.88 ± 2.42	0.171
39	Coagulation factor X:Homo sapiens	7.11 ± 2.43	0.170
40	Sodium channel protein type IX alpha subunit:Homo sapiens	6.43 ± 2.43	0.170
41	Cholecystokinin B receptor:Homo sapiens	6.61 ± 2.43	0.169
42	Matrix metalloproteinase-2:Homo sapiens	6.88 ± 2.43	0.169
43	Neuropeptide Y receptor type 5:Rattus norvegicus	7.78 ± 2.43	0.169
44	Matrix metalloproteinase 13:Homo sapiens	7.67 ± 2.43	0.169
45	P2X purinoceptor 7:Homo sapiens	7.14 ± 2.44	0.169
46	Poly [ADP-ribose] polymerase 14:Homo sapiens	5.84 ± 2.44	0.169
47	Histone deacetylase 1:Homo sapiens	6.69 ± 2.44	0.168
48	Monoglyceride lipase:Homo sapiens	6.36 ± 2.44	0.168
49	Muscarinic acetylcholine receptor M2:Homo sapiens	6.77 ± 2.44	0.168
50	Rho-associated protein kinase 2:Homo sapiens	7.35 ± 2.44	0.168
51	c-Jun N-terminal kinase 3:Homo sapiens	6.38 ± 2.44	0.168
52	Cholinesterase:Equus caballus	5.72 ± 2.45	0.167

Position	Target	pIC50	τ
53	Beta-secretase 1:Homo sapiens	6.68 ± 2.45	0.167
54	Muscarinic acetylcholine receptor M1:Homo sapiens	6.68 ± 2.45	0.167
55	Nicotinamide phosphoribosyltransferase:Homo sapiens	7.42 ± 2.45	0.167
56	Serotonin 2b (5-HT2b) receptor:Homo sapiens	6.63 ± 2.45	0.167
57	Matrix metalloproteinase-1:Homo sapiens	6.26 ± 2.45	0.167
58	LIM domain kinase 2:Homo sapiens	7.62 ± 2.45	0.167
59	Neuropeptide Y receptor type 2:Homo sapiens	6.06 ± 2.45	0.166
60	c-Jun N-terminal kinase 1:Homo sapiens	6.69 ± 2.45	0.166
61	Dopamine D2 receptor:Rattus norvegicus	6.75 ± 2.45	0.166
62	Calcitonin gene-related peptide type 1 receptor:Homo sapiens	7.56 ± 2.45	0.166
63	Neuropeptide Y receptor type 5:Mus musculus	8.13 ± 2.46	0.166
64	Orexin receptor 1:Homo sapiens	6.85 ± 2.46	0.166
65	Orexin receptor 2:Homo sapiens	7.00 ± 2.46	0.166
66	Butyrylcholinesterase:Mus musculus	6.45 ± 2.46	0.166
67	Neurokinin 1 receptor:Homo sapiens	7.92 ± 2.46	0.166
68	Serine/threonine-protein kinase AKT:Homo sapiens	6.97 ± 2.46	0.165
69	Alkaline phosphatase, tissue-nonspecific isozyme:Homo sapiens	5.06 ± 2.46	0.165
70	Serine/threonine-protein kinase Chk1:Homo sapiens	7.41 ± 2.46	0.165
71	Matrix metalloproteinase 8:Homo sapiens	7.49 ± 2.46	0.165
72	Vanilloid receptor:Rattus norvegicus	6.82 ± 2.46	0.165
73	Acetylcholinesterase:Electrophorus electricus	5.98 ± 2.46	0.165
74	Matrix metalloproteinase 3:Homo sapiens	6.75 ± 2.46	0.165
75	ADAM17:Sus scrofa	8.11 ± 2.46	0.165
76	Sodium/calcium exchanger 1:Homo sapiens	6.27 ± 2.46	0.165
77	Ghrelin receptor:Homo sapiens	7.24 ± 2.46	0.165
78	Sodium channel protein type V alpha subunit:Homo sapiens	5.57 ± 2.46	0.165
79	Tankyrase-1:Homo sapiens	6.86 ± 2.47	0.164

Position	Target	pIC50	τ
80	Cholecystokinin A receptor:Rattus norvegicus	6.35 ± 2.47	0.164
81	Phosphodiesterase 10A:Homo sapiens	7.83 ± 2.47	0.164
82	Prostanoid DP receptor:Mus musculus	7.89 ± 2.47	0.164
83	Protein farnesyltransferase:Homo sapiens	6.95 ± 2.47	0.164
84	Transforming protein RhoA:Homo sapiens	4.93 ± 2.47	0.164
85	DNA-dependent protein kinase:Homo sapiens	6.38 ± 2.47	0.164
86	Muscarinic acetylcholine receptor M5:Homo sapiens	6.03 ± 2.47	0.164
87	Mu opioid receptor:Homo sapiens	6.59 ± 2.47	0.164
88	Delta opioid receptor:Homo sapiens	7.28 ± 2.47	0.163
89	Acetylcholinesterase:Mus musculus	6.35 ± 2.47	0.163
90	Mitochondrial import inner membrane translocase subunit TIM10:Saccharomyces cerevisiae S288c	4.69 ± 2.48	0.163
91	Tyrosine-protein kinase receptor FLT3:Homo sapiens	6.93 ± 2.48	0.163
92	ADAM17:Homo sapiens	6.97 ± 2.48	0.163
93	Alpha-1,6-mannosyl-glycoprotein 2-beta-N-acetylglucosaminyltransferase:Homo sapiens	7.17 ± 2.48	0.163
94	Melanin-concentrating hormone receptor 1:Mus musculus	7.16 ± 2.48	0.163
95	Sodium-dependent proline transporter:Homo sapiens	6.54 ± 2.48	0.163
96	Tyrosine-protein kinase TYK2:Homo sapiens	6.71 ± 2.48	0.163
97	Smoothened homolog:Homo sapiens	6.87 ± 2.48	0.163
98	Integrin alpha-V/beta-3:Homo sapiens	7.65 ± 2.48	0.163
99	Membrane-bound transcription factor site-1 protease:Homo sapiens	6.43 ± 2.48	0.163
100	Butyrylcholinesterase:Homo sapiens	6.08 ± 2.48	0.162

2. Inhibition mechanism profiles

Figure S1. Inhibition kinetics and Lineweaver-Burk plot (r^2 = 0.996) for *ee*AChE (0.09 U/mL) and **1** (0-500 nM) by using different substrate (acetylthiocholine iodide) concentrations (50-300 μM). The replot (r^2 = 0.995) of the slopes versus [I] determined the K_i (56 nM) as the *x*-axis intercept. (○) no inhibitor, (●) 50 nM, (■) 100 nM, (◆) 200 nM, (▲) 500 nM.

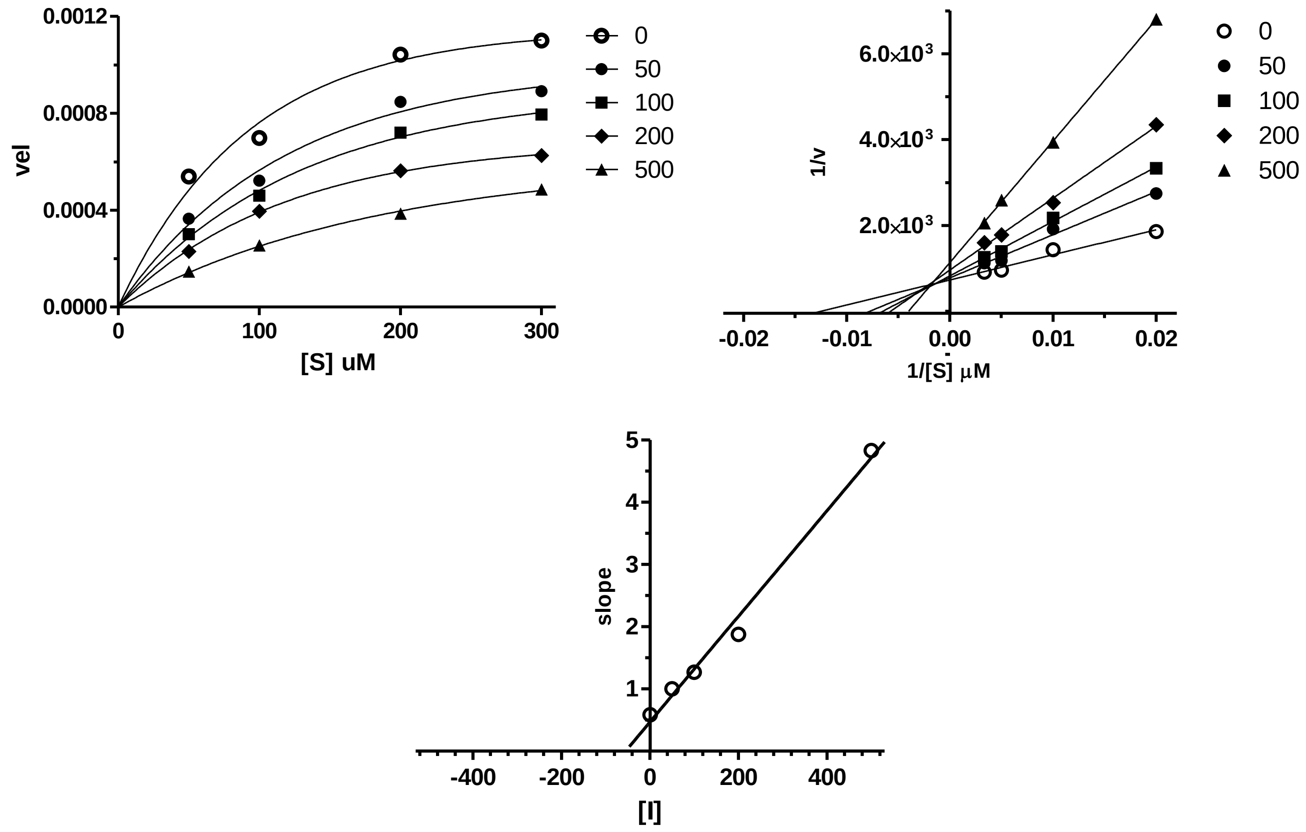

Figure S2. Inhibition kinetics and Lineweaver-Burk plot (r^2 = 0.964-0.996) for eqBChE (0.18 U/mL) and **14** (0-1 μM) by using different substrate (butyrylthiocholine iodide) concentrations (50-300 μM). The replot (r^2 = 0.9914) of the slopes versus [I] determined the Ki (104 nM) as the x-axis intercept. (○) no inhibitor, (●) 50 nM, (■) 100 nM, (◆) 200 nM, (▲) 500 nM, (▼) 1 μM.

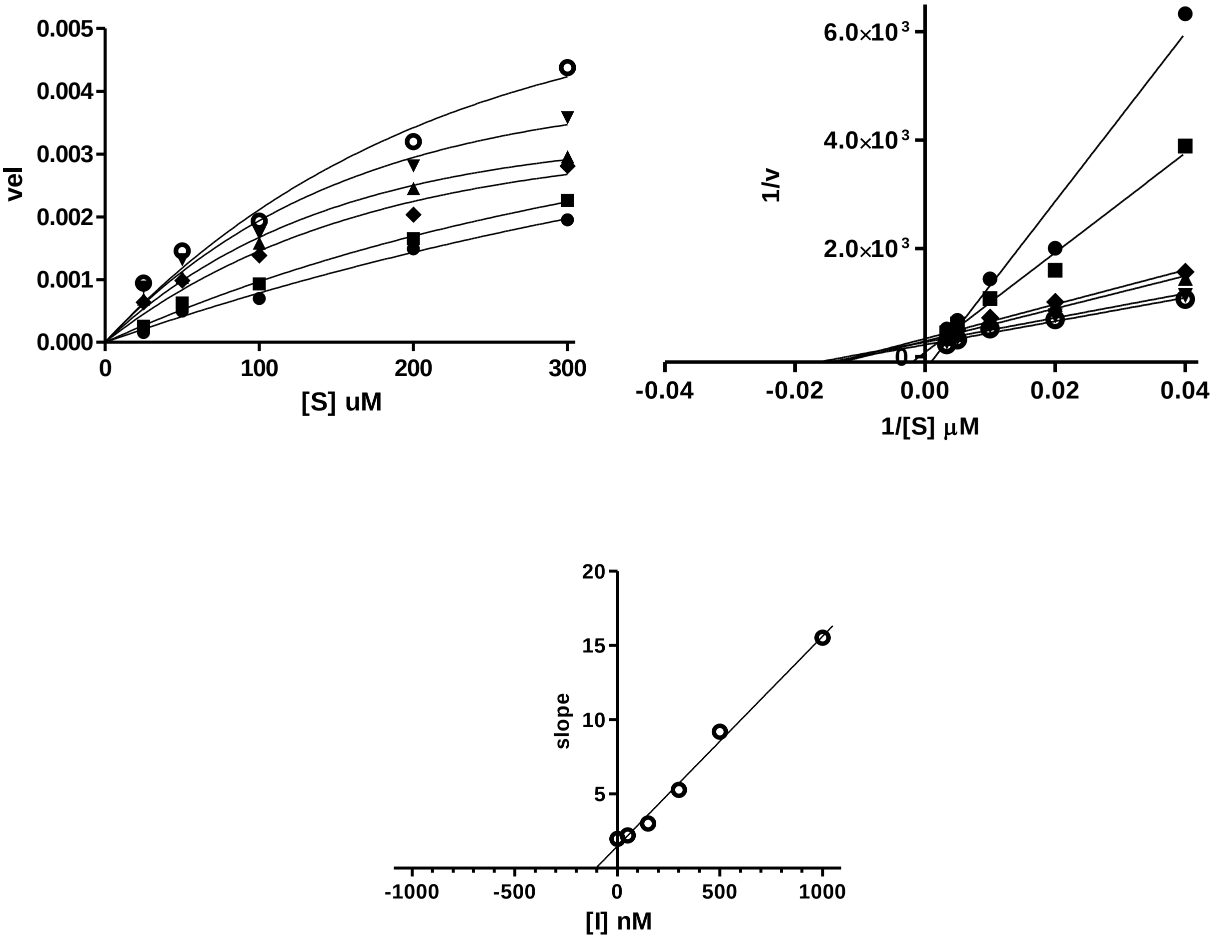

3. Molecular modelling

Table S1.
Similarity matrix based on four different MIFs computed for BChE and FXa with BioGPS.

	H	CRY	N1	O
**H**	0.75	0.74	0.74	0.71
**CRY**	0.74	0.75	0.74	0.72
**N1**	0.74	0.74	0.73	0.71
**O**	0.71	0.72	0.71	0.70

Table S2.
Similarity matrix based on four different MIFs computed for of AChE and thrombin with BioGPS.

	H	CRY	N1	O
**H**	0.77	0.75	0.76	0.75
**CRY**	0.75	0.75	0.75	0.73
**N1**	0.76	0.75	0.77	0.75
**O**	0.75	0.73	0.75	0.75

4. 1H NMR spectra

Compound **9**

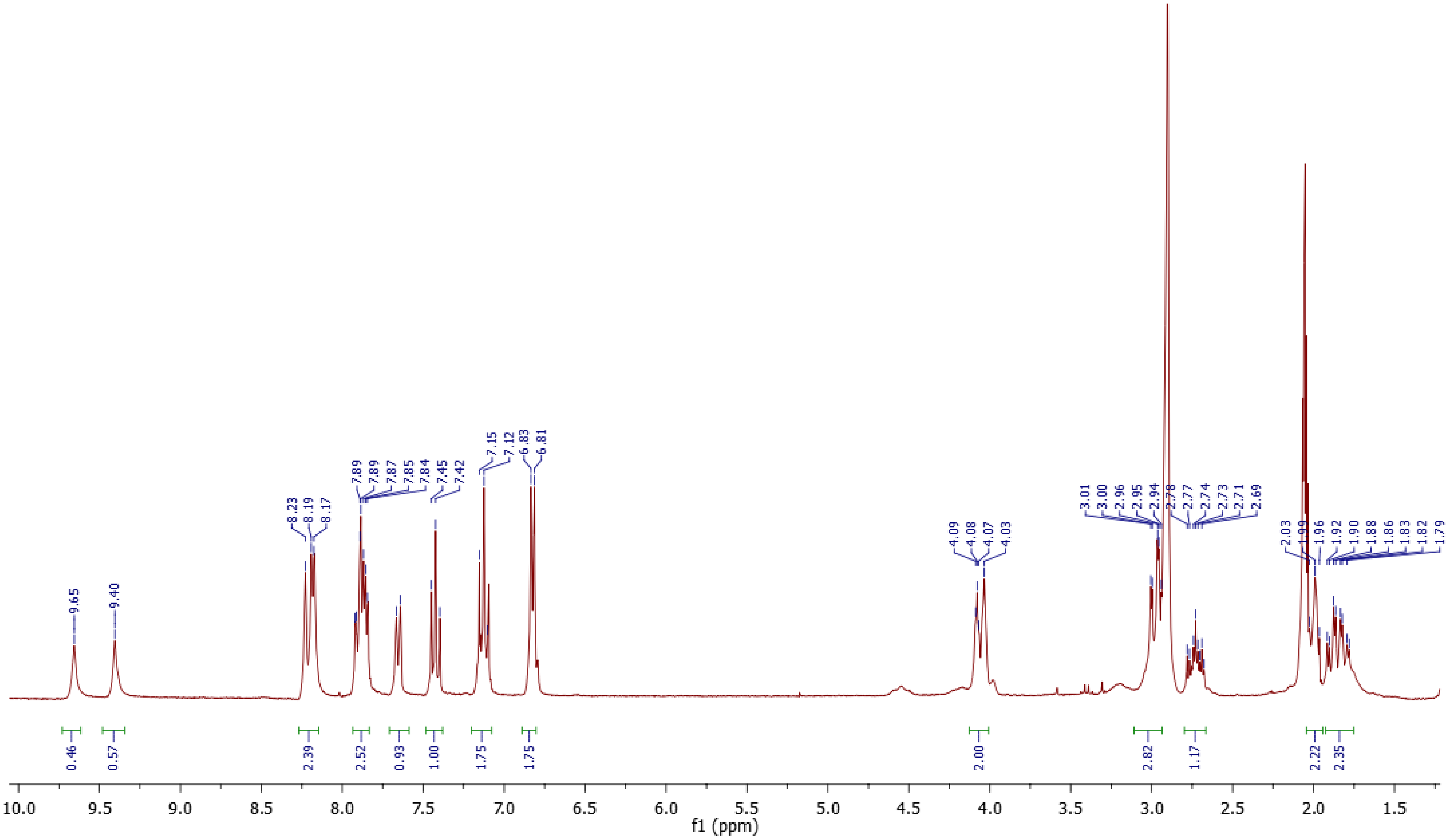

Compound **10**

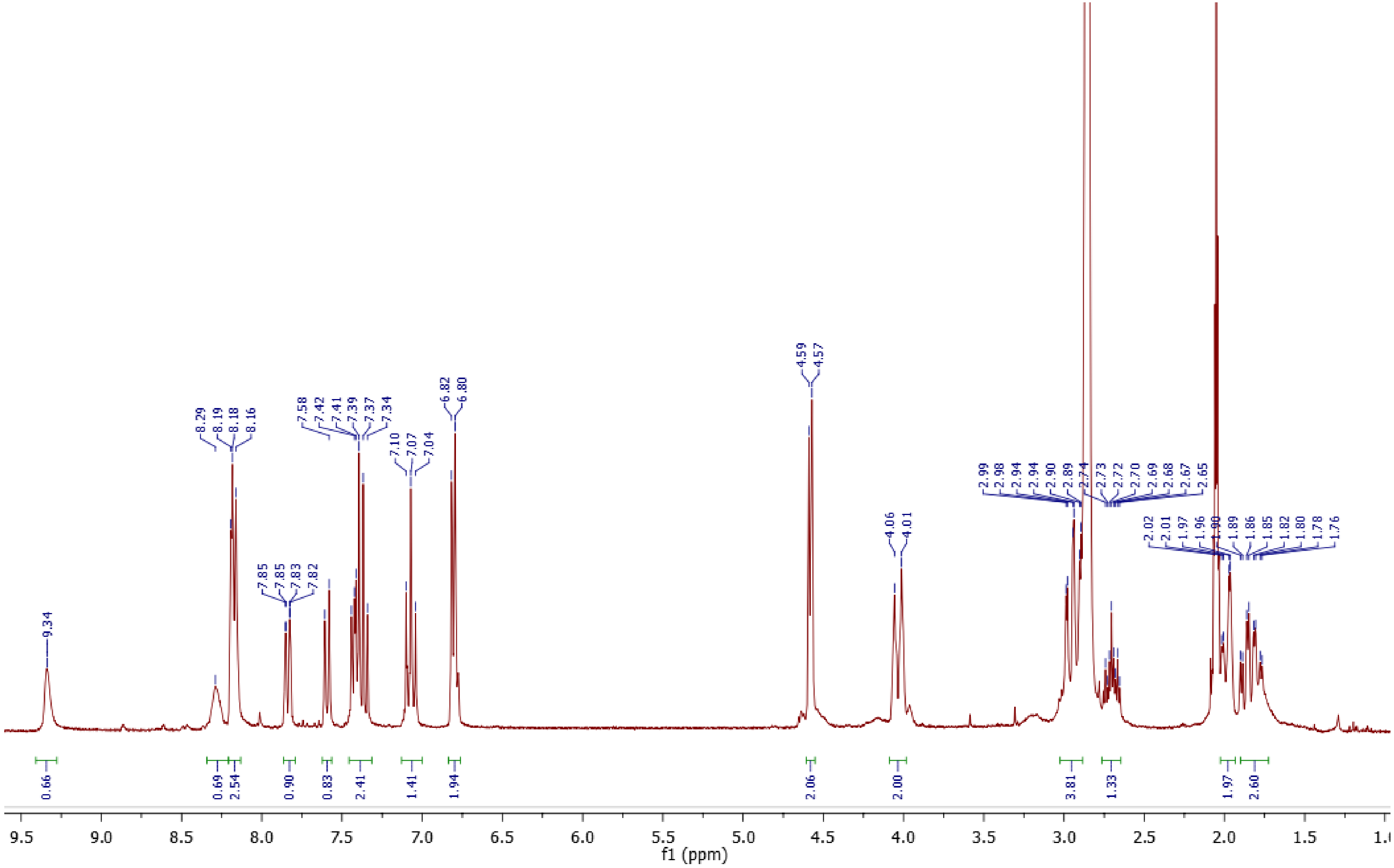

Compound **11**

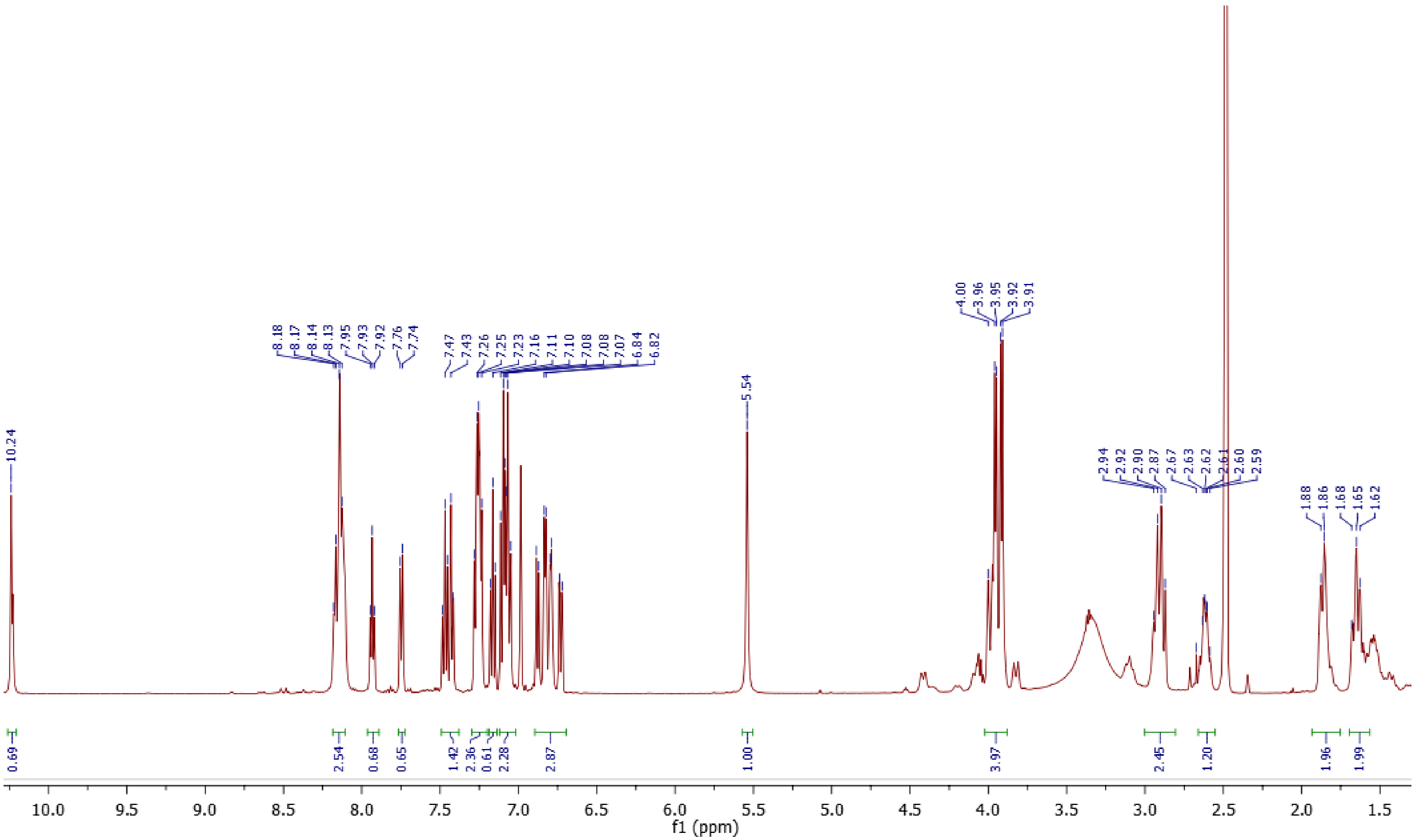
10.24
8.18
8.17
8.14
8.13
7.95
7.93
7.92
7.76
7.74
7.47
7.43
7.26
7.25
7.23
7.16
7.11
7.10
7.08
7.08
7.07
6.84
6.82
5.54
4.00
3.96
3.95
3.92
3.91
2.94
2.92
2.90
2.87
2.67
2.63
2.62
2.61
2.60
2.59
1.88
1.86
1.68
1.65
1.62
0.69
2.54
0.68
0.65
1.42
2.36
0.61
2.28
2.87
1.00
3.97
2.45
1.20
1.96
1.99
10.0
9.5
9.0
8.5
8.0
7.5
7.0
6.5
6.0
5.5
5.0
4.5
4.0
3.5
3.0
2.5
2.0
1.5
f1 (ppm)

Compound **12**

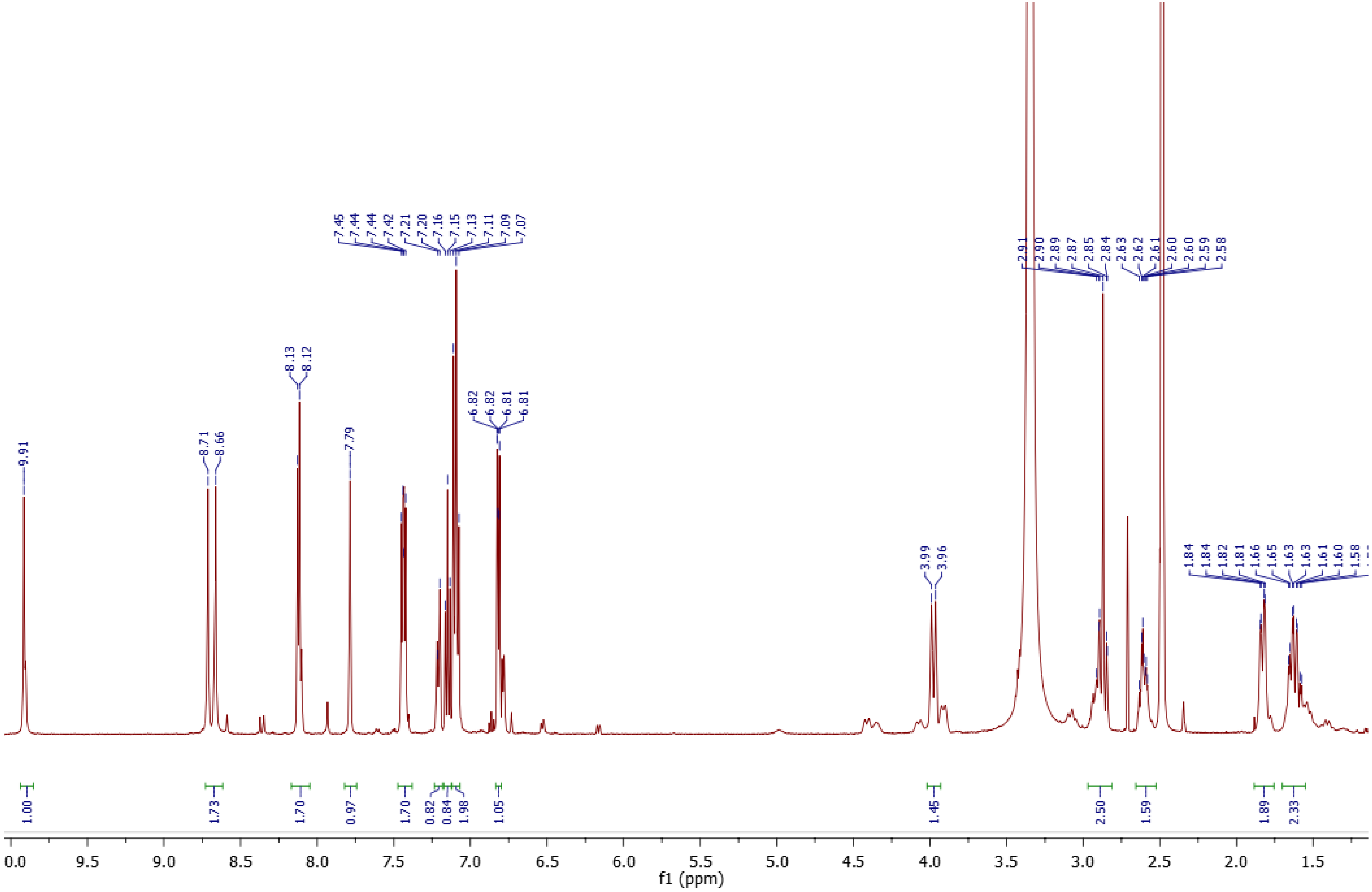

Compound **14**

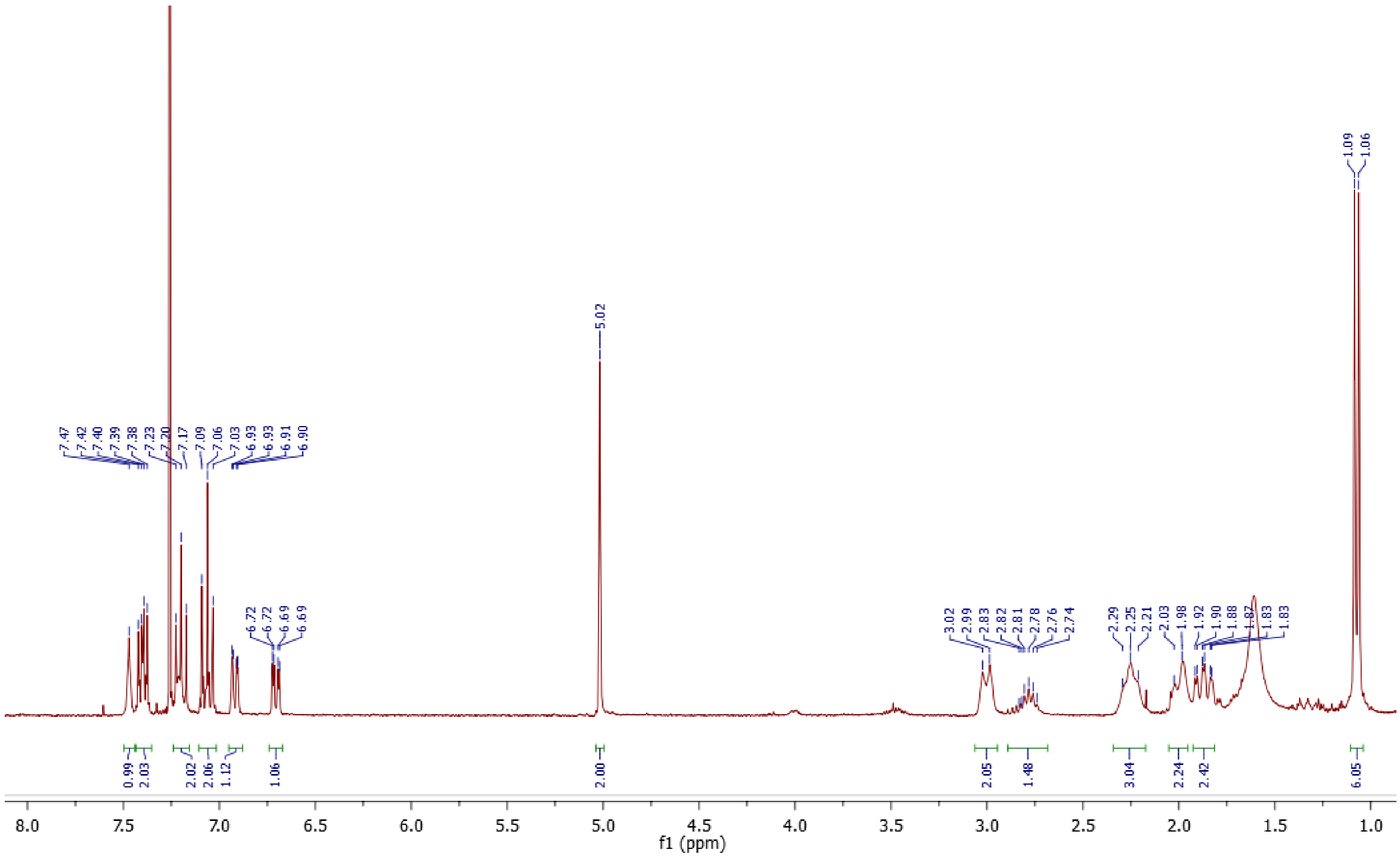

Compound **15**

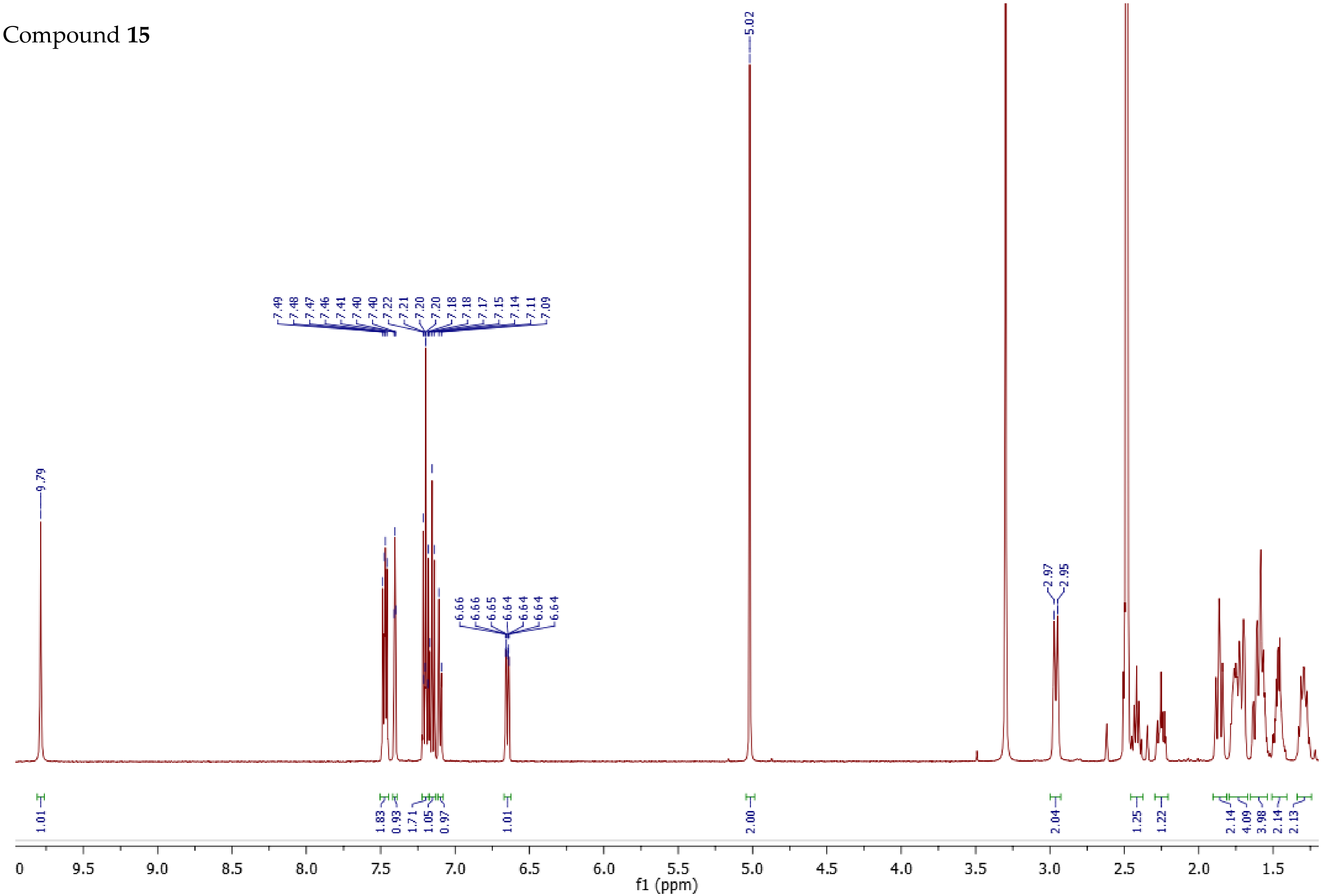
9.79
7.49 7.48 7.47 7.46 7.41 7.40 7.40 7.22 7.21 7.20 7.20 7.18 7.18 7.17 7.15 7.14 7.11 7.09
6.66 6.66 6.65 6.64 6.64 6.64 6.64
5.02
2.97 2.95
1.01
1.83 0.93 1.71 1.05 0.97
1.01
2.00
2.04
1.25 1.22
2.14 4.09 3.98 2.14 2.13
0 9.5 9.0 8.5 8.0 7.5 7.0 6.5 6.0 5.5 5.0 4.5 4.0 3.5 3.0 2.5 2.0 1.5
f1 (ppm)

Compound **16**

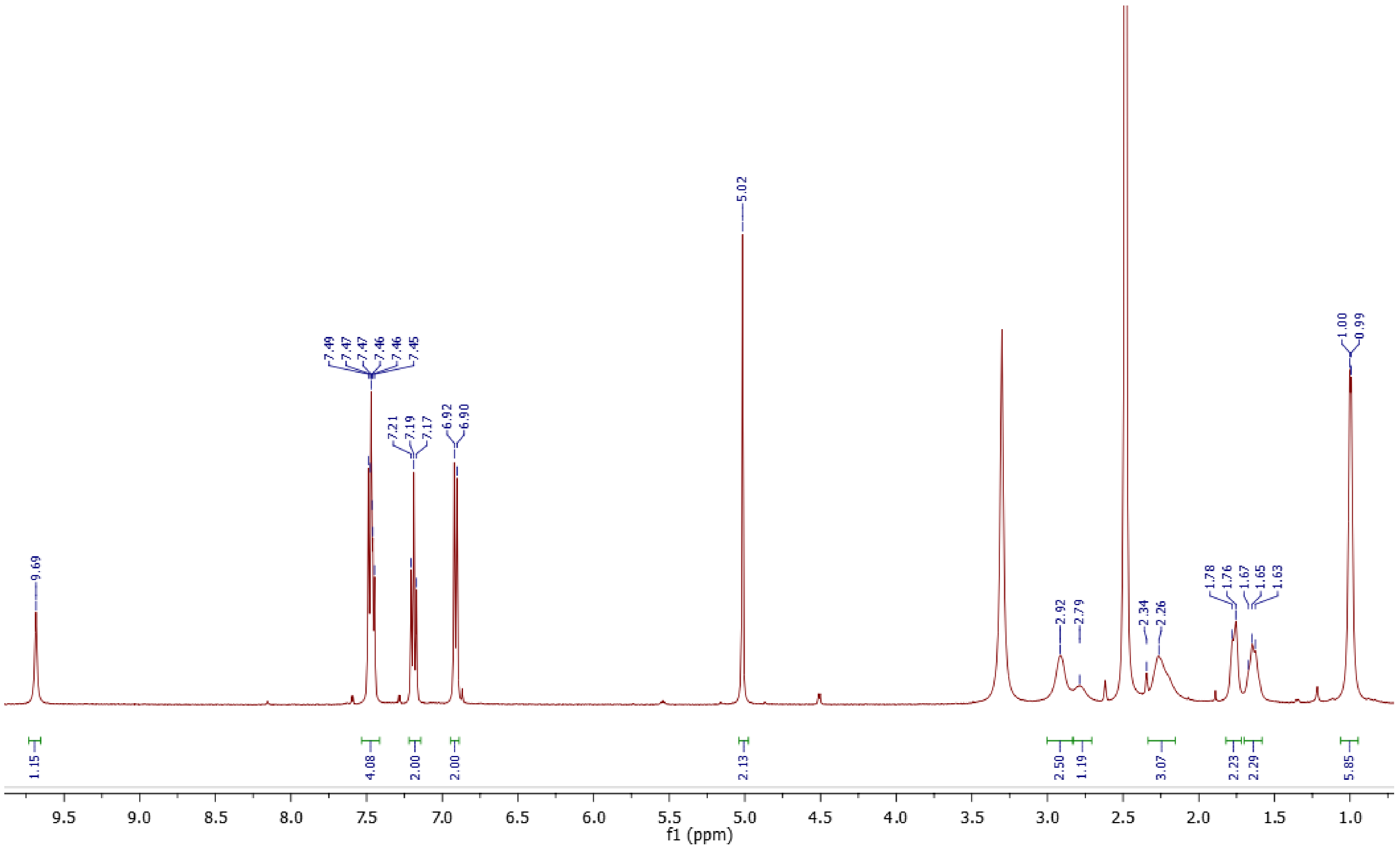
9.69
7.49
7.47
7.47
7.46
7.46
7.45
7.21
7.19
7.17
6.92
6.90
5.02
2.92
2.79
2.34
2.26
1.78
1.76
1.67
1.65
1.63
1.00
0.99
1.15
4.08
2.00
2.00
2.13
2.50
1.19
3.07
2.23
2.29
5.85
9.5
9.0
8.5
8.0
7.5
7.0
6.5
6.0
5.5
5.0
4.5
4.0
3.5
3.0
2.5
2.0
1.5
1.0
f1 (ppm)

Compound **17**

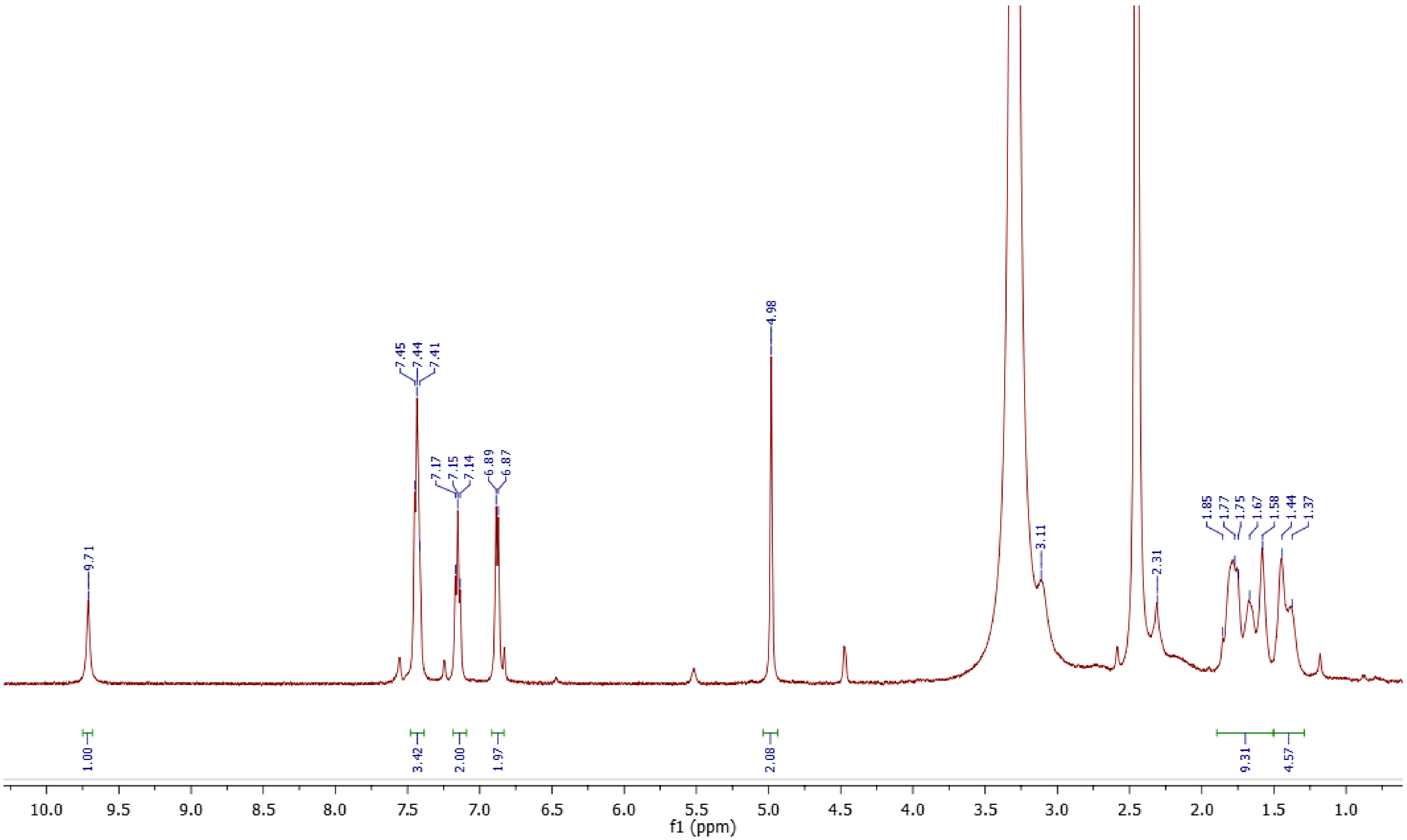
9.71
7.45
7.44
7.41
7.17
7.15
7.14
6.89
6.87
4.98
3.11
2.31
1.85
1.77
1.75
1.67
1.58
1.44
1.37
1.00
3.42
2.00
1.97
2.08
9.31
4.57
10.0
9.5
9.0
8.5
8.0
7.5
7.0
6.5
6.0
5.5
5.0
4.5
4.0
3.5
3.0
2.5
2.0
1.5
1.0
f1 (ppm)

Compound **18**

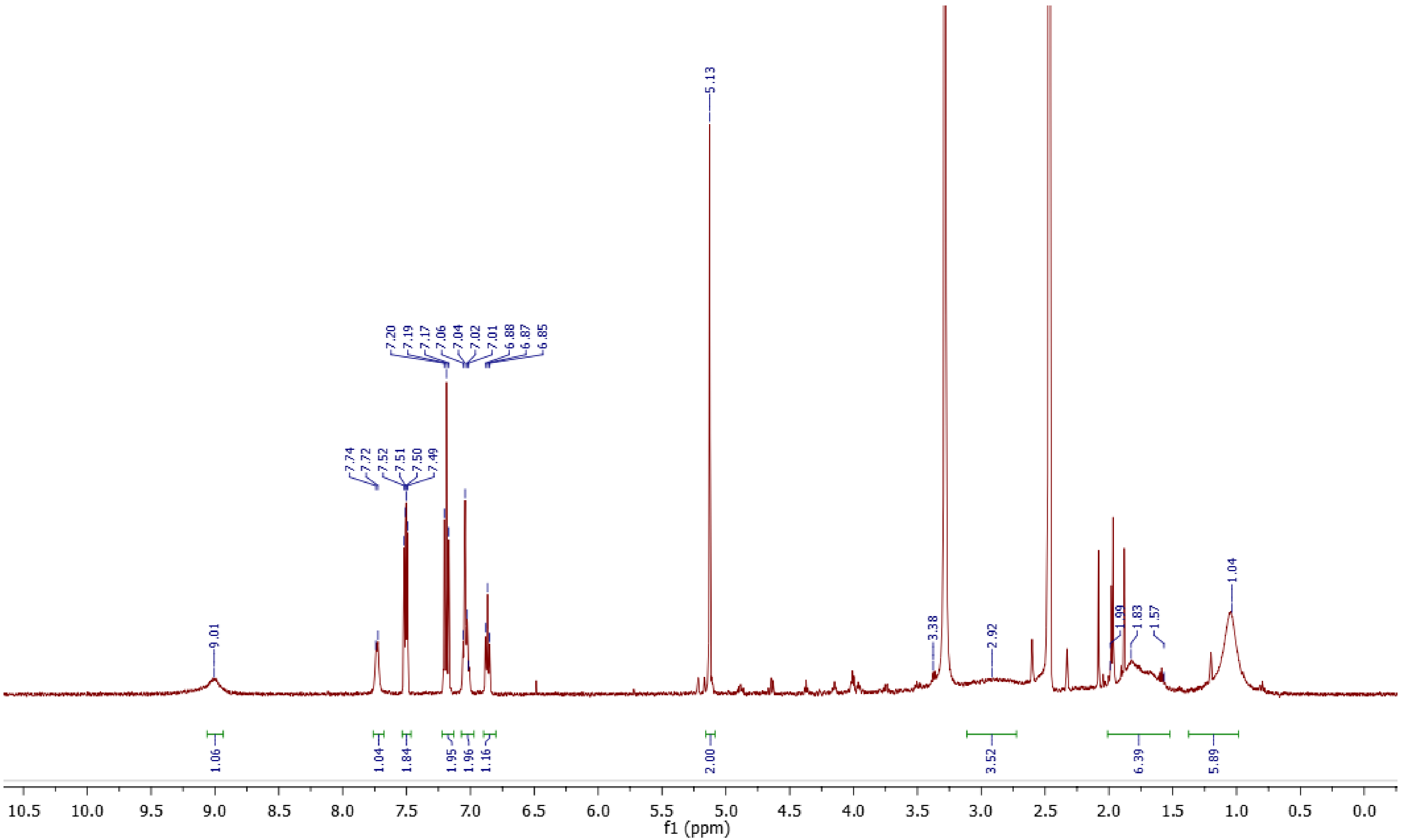

7.74
7.72
7.52
7.51
7.50
7.49
7.20
7.19
7.17
7.06
7.04
7.02
7.01
6.88
6.87
6.85
9.01
5.13
3.38
2.92
1.99
1.83
1.57
1.04
1.06
1.04
1.84
1.95
1.96
1.16
2.00
3.52
6.39
5.89
10.5
10.0
9.5
9.0
8.5
8.0
7.5
7.0
6.5
6.0
5.5
5.0
4.5
4.0
3.5
3.0
2.5
2.0
1.5
1.0
0.5
0.0
f1 (ppm)

Compound **20**

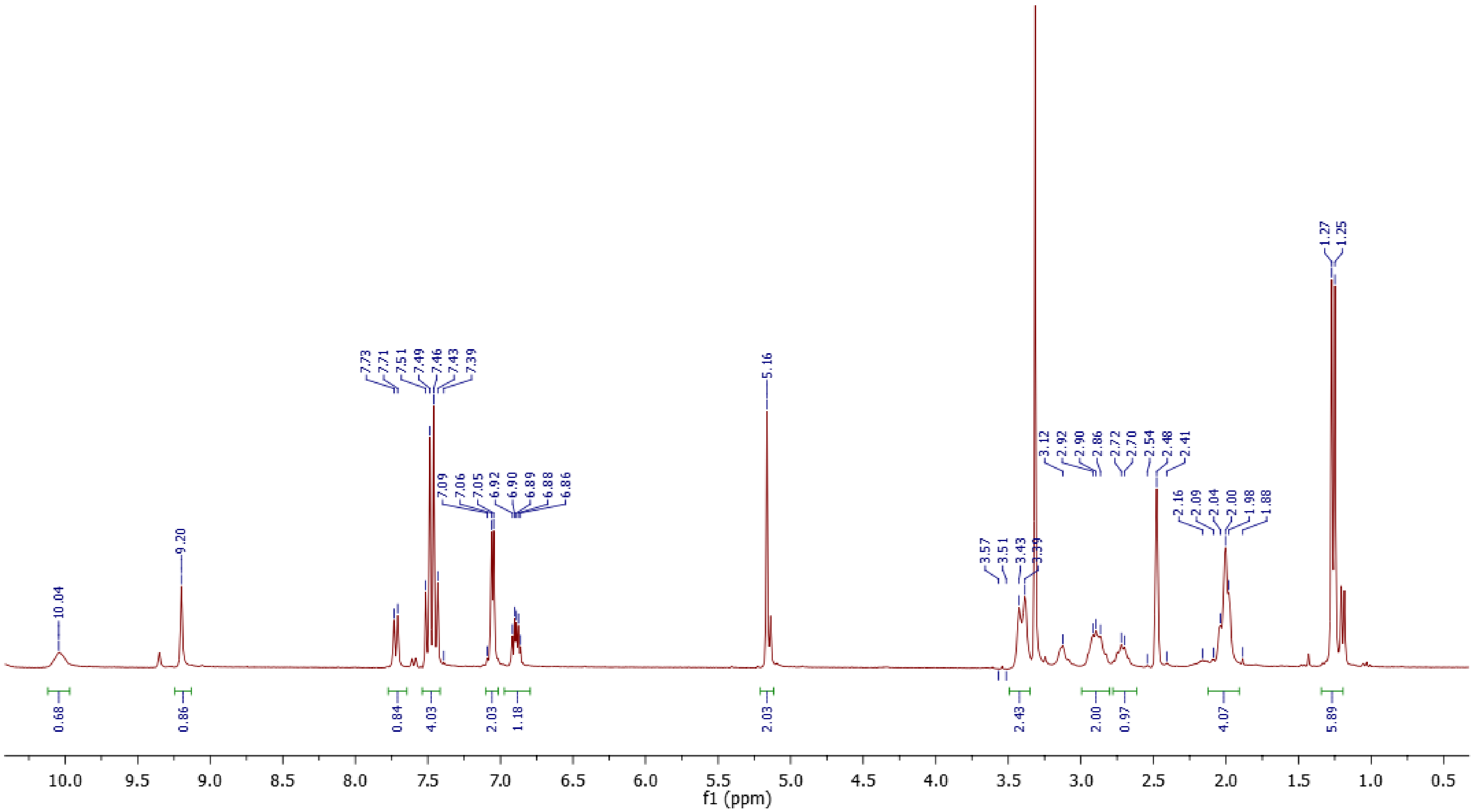

Compound **21**

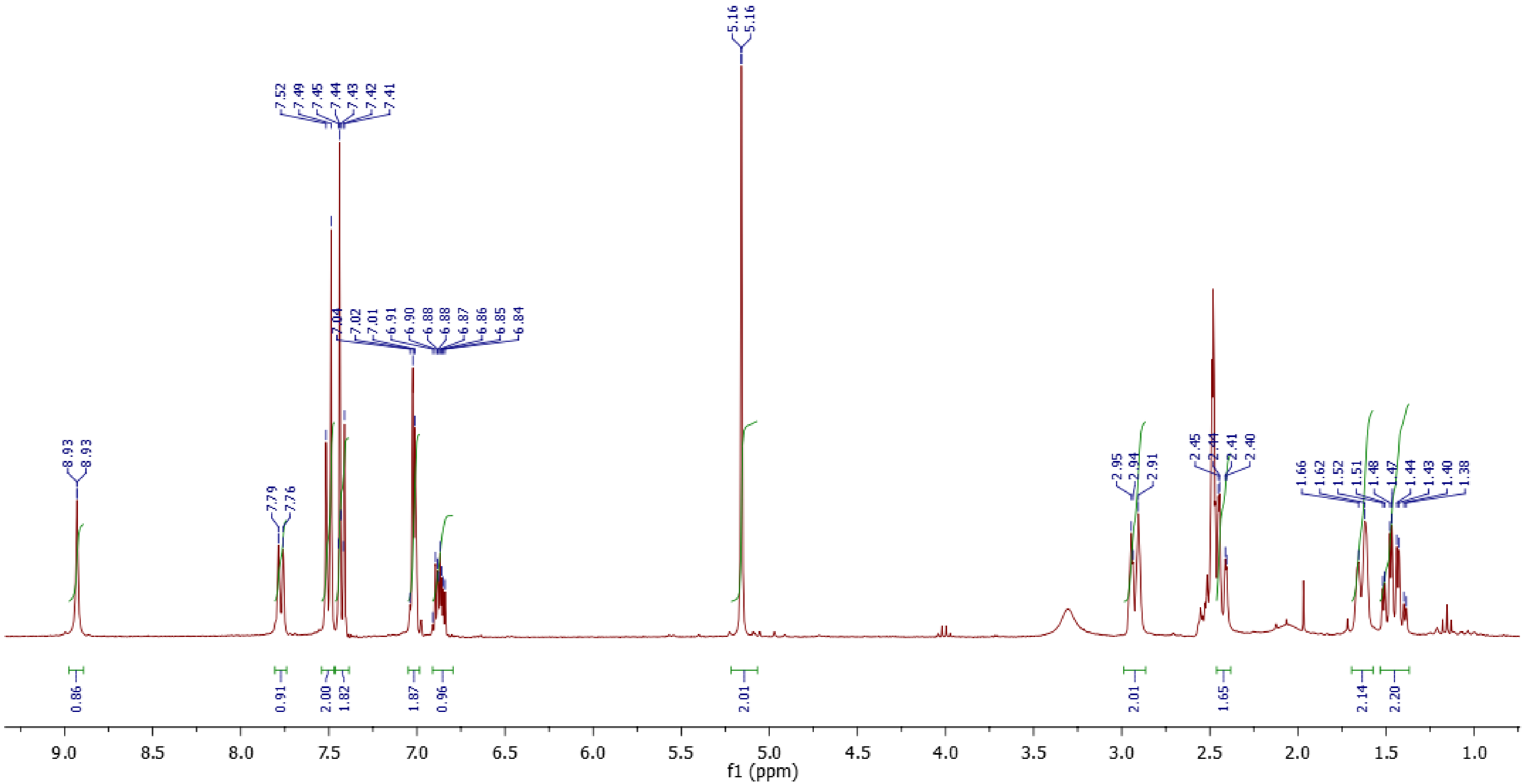
8.93
8.93
7.79
7.76
7.52
7.49
7.45
7.44
7.43
7.42
7.41
7.04
7.02
7.01
6.91
6.90
6.88
6.88
6.87
6.86
6.85
6.84
5.16
5.16
2.95
2.94
2.91
2.45
2.44
2.41
2.40
1.66
1.62
1.52
1.51
1.48
1.47
1.44
1.43
1.40
1.38
0.86
0.91
2.00
1.82
1.87
0.96
2.01
2.01
1.65
2.14
2.20
9.0
8.5
8.0
7.5
7.0
6.5
6.0
5.5
5.0
4.5
4.0
3.5
3.0
2.5
2.0
1.5
1.0
f1 (ppm)

Compound **28**

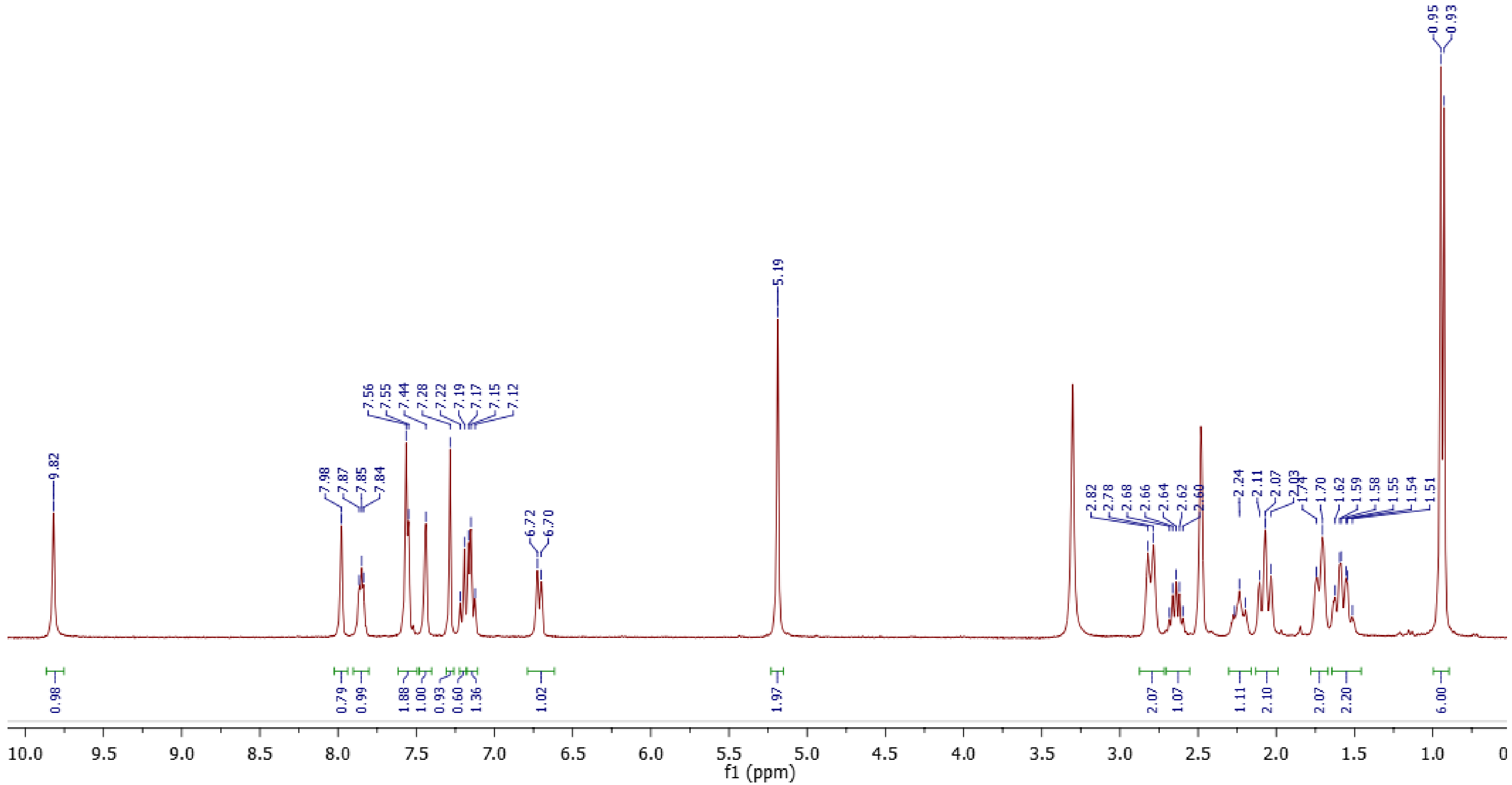

Supplement: Supplementary file 1 [file molecules-26-05208-s001.zip › molecules-1344480-supplementary.PDF]
